# Long-term spironolactone treatment reduces coronary TRPC expression, vasoconstriction, and atherosclerosis in metabolic syndrome pigs

**DOI:** 10.1007/s00395-017-0643-0

**Published:** 2017-07-29

**Authors:** Wennan Li, Xingjuan Chen, Ashley M. Riley, S. Christopher Hiett, Constance J. Temm, Eleni Beli, Xin Long, Saikat Chakraborty, Mouhamad Alloosh, Fletcher A. White, Maria B. Grant, Michael Sturek, Alexander G. Obukhov

**Affiliations:** 1 Department of Cellular and Integrative Physiology, Indiana University School of Medicine, Indianapolis, 635 Barnhill Dr., MS360A, Indianapolis, IN 46202 USA; 2 Indiana University Pathology, Immunohistochemistry Core, Indiana University School of Medicine, Indianapolis, Indianapolis, IN USA; 3 Department of Anesthesia, Indiana University School of Medicine, Indianapolis, Indianapolis, IN USA; 4 Department of Ophthalmology, Indiana University School of Medicine, Indianapolis, Indianapolis, IN USA

**Keywords:** Coronary artery, Metabolic syndrome, Atherosclerosis, Spironolactone, TRPC

## Abstract

Coronary transient receptor potential canonical (TRPC) channel expression is elevated in metabolic syndrome (MetS). However, differential contribution of TRPCs to coronary pathology in MetS is not fully elucidated. We investigated the roles of TRPC1 and TRPC6 isoforms in coronary arteries of MetS pigs and determined whether long-term treatment with a mineralocorticoid receptor inhibitor, spironolactone, attenuates coronary TRPC expression and associated dysfunctions. MetS coronary arteries exhibited significant atherosclerosis, endothelial dysfunction, and increased histamine-induced contractions. Immunohistochemical studies revealed that TRPC6 immunostaining was significantly greater in the medial layer of MetS pig coronary arteries compared to that in Lean pigs, whereas little TRPC6 immunostaining was found in atheromas. Conversely, TRPC1 immunostaining was weak in the medial layer but strong in MetS atheromas, where it was predominantly localized to macrophages. Spironolactone treatment significantly decreased coronary TRPC expression and dysfunctions in MetS pigs. In vivo targeted delivery of the dominant-negative (DN)-TRPC6 cDNA to the coronary wall reduced histamine-induced calcium transients in the MetS coronary artery medial layer, implying a role for TRPC6 in mediating calcium influx in MetS coronary smooth muscles. Monocyte adhesion was increased in Lean pig coronary arteries cultured in the presence of aldosterone; and spironolactone antagonized this effect, suggesting that coronary mineralocorticoid receptor activation may regulate macrophage infiltration. TRPC1 expression in atheroma macrophages was associated with advanced atherosclerosis, whereas medial TRPC6 upregulation correlated with increased histamine-induced calcium transients and coronary contractility. We propose that long-term spironolactone treatment may be a therapeutic strategy to decrease TRPC expression and coronary pathology associated with MetS.

**Electronic supplementary material:**

The online version of this article (doi:10.1007/s00395-017-0643-0) contains supplementary material, which is available to authorized users.

## Introduction

Metabolic syndrome (MetS) is a condition characterized by central obesity, insulin resistance, glucose intolerance, dyslipidemia, hypertension, and atherosclerosis [23, 38, 50]. Atherosclerosis usually occurs in the large and medium-sized arteries [19]. Atherosclerotic plaques are made of lipids, cholesterol, extracellular matrix deposits, proliferating smooth muscle cells (SMCs), T-lymphocytes, foam cells, and macrophages. These lesions decrease the vessel lumen area and cause obstruction of blood flow. Furthermore, atherosclerotic coronary arteries exhibit increased spasticity [46, 49] that may lead to angina, myocardial infarction, and sudden death [27].

Angina patients present with elevated plasma histamine concentration [43], indicating that histamine-induced coronary artery contractions may contribute to some ischemic episodes in these patients. Indeed, histamine is known to contract coronary arteries, with the presence of atherosclerosis potentiating the contractile response [11, 22]. In the coronary circulation, histamine mainly originates from mast cells infiltrating atherosclerotic lesions [47]; and mast cells are often found at the site of coronary spasm in the patients with angina [18]. In turn, histamine is an activator of transient receptor potential canonical (TRPC) calcium-permeable cation channels [10, 12, 41], which are expressed in vascular SMCs. TRPC channels are implicated in the pathogenesis of hypertension and neointimal hyperplasia [1, 33, 40].

One of the features of MetS is elevated plasma aldosterone [6, 13, 32]. Aldosterone is an agonist of the mineralocorticoid receptor, which is widely expressed in the kidney, heart, and vasculature [29, 37]. Elevated aldosterone may induce extracellular matrix remodeling by enhancing collagen synthesis, fibrosis, and vascular inflammation [5, 36] and is considered a risk factor for heart failure, myocardial infarction, stroke, and coronary artery disease [25, 28, 35]. Indeed, both human and animal studies have shown that aldosterone may be involved in facilitating atherosclerosis development [15, 20, 25, 31, 37].

Our previous study showed that TRPC1 and TRPC6 expression is increased in coronary arteries from MetS pigs as compared to Lean pigs [16]. We also reported that aldosterone, acting via the mineralocorticoid receptor, increased TRPC1 and TRPC6 expression in the adrenal medulla of MetS pigs [26] exhibiting elevated plasma aldosterone [2]. Consistently, Bae et al. reported that mineralocorticoids enhance TRPC6 expression in rat aortic smooth muscle cells [3]. In this study, we investigated the roles of TRPC1 and TRPC6 channels in the coronary artery of MetS pigs and whether long-term inhibition of the mineralocorticoid receptor by spironolactone downregulates coronary TRPC expression and attenuates MetS-induced coronary pathology.

## Materials and methods

### Animals

Thirty-six castrated Ossabaw miniature pigs were subdivided into three groups: Lean, metabolic syndrome (MetS), and metabolic syndrome treated with spironolactone (MetS-SN). The pigs were initially fed with the standard chow consisting of 22% kcal from protein, 70% kcal from carbohydrates, and 8% kcal from fat (5L80 diet, Purina Test Diet, Richmond, IN, USA) until an age of 6–8 months. Then, the Lean group of pigs was maintained on the same standard chow for an additional 7–9 months. The MetS group of pigs was fed with an atherogenic diet, consisting of 16% kcal from protein, 21% kcal from mixed carbohydrates, 20% kcal from fructose, and 43% kcal from fat, which was supplemented with 2% cholesterol and 0.7% sodium cholate by weight (KT324 diet, Purina Test Diet, Richmond, IN, USA; 1 kg/day) for 7–9 months, similar to previous reports [16]. The pigs from the MetS-SN group were fed the atherogenic KT324 diet (1 kg/day) mixed with spironolactone (3 mg/kg body weight/day). During this study, two out of 13 investigated MetS pigs prematurely died. Postmortem histological assessment indicated that these two pigs had very advanced atherosclerosis, suggesting that the pigs had likely died due to a myocardial infarction. In contrast, no deaths occurred in the Lean (14 pigs) or MetS-SN (9 pigs) groups.

### Intravenous glucose tolerance test (IVGTT)

Prior to the test, pigs were acclimated to a canvas sling, which was used to suspend a pig above the ground, for 30–60 min daily for 3–5 days. A day before an IVGTT, the central venous catheter (Cook Medical, Bloomington, IN, USA) was percutaneously placed into the jugular vein under isoflurane anesthesia and secured on the lateral side of the neck for future blood sampling. All pigs were fasted overnight before IVGTTs, and the tests were performed without anesthesia because it artificially elevates blood glucose concentration. A single dose of 1 g glucose/kg body weight was given at a time of zero through the central venous catheter. Then, ~3 ml blood samples were collected every 10 min for 60 min via the same catheter to assess glucose tolerance. Blood samples were centrifuged at 2000 rpm for 20 min at 4 °C to collect blood serum; serum glucose concentration was determined using an YSI 2300 STAT plus Glucose analyzer (YSI Life Sciences., Yellow Springs, OH, USA).

### Intravascular ultrasound (IVUS)

Ceftiofur (5 mg/kg, IM) and aspirin (300 mg) were given to pigs on the day of the procedure. Anesthesia induction was carried out using an intramuscular injection of a mixture of telazol (5 mg/kg) and xylazine (3 mg/kg) that was followed by isoflurane endotracheally (2–4% mixed with 100% O_2_) to maintain anesthesia. The anesthetized, heparinized, and intubated unconscious animal was secured in a supine position, and a subcutaneous injection of a mixture of topical anesthetics (2% lidocaine and 2% bupivacaine; 50:50) was given before the surgery. The arterial sheath introducer (7 F introducer sheath, Boston Scientific, Marlborough, MA, USA) was placed into the right femoral artery through an arteriotomy. Using X-ray angiography to monitor the progress of placements, the guiding catheter (7 F guide catheter, Mach1, HS SH, AL.75 SH, Boston Scientific) engaged the coronary ostium and then an angioplasty guidewire was deployed into a coronary artery. A contrast solution injection (OMNIPAQUE, GE healthcare, Princeton, NJ, USA) was used to visualize the coronary artery tree. Then, the Revolution 45 MHz Rotational Imaging Intravascular Ultrasound Catheter-transducer (Volcano Inc, Rancho Cordova, CA, USA) was advanced along the guide wire to the distal part of the coronary artery and the pullback of the IVUS transducer was performed at 0.5 mm/s. Cross-sectional IVUS images were acquired every 2 mm through the ~40 mm length of coronary arteries. The plaque wall coverage was measured using the Image-Pro Premier 9.1 software package (Media Cybernetics Inc., Warrendale, PA, USA) to determine the extent of atherosclerosis. Percent wall coverage (% wall coverage) was calculated as the length of plaque-covered coronary artery wall segment divided by the coronary artery perimeter and multiplied by 100. The wall coverage data from 20 images for each individual right coronary artery (RCA) and left anterior descending (LAD) coronary artery of a pig were averaged to represent the mean wall coverage value for the pig. Then the mean wall coverage for each treatment group was calculated by averaging the mean wall coverage data obtained in each pig.

### Masson’s trichrome staining and immunohistochemistry

Freshly isolated coronary arteries were cut into 3 mm cross-sectioned rings, fixed in 10% phosphate-buffered formalin (Fisher Scientific, Fair Lawn, NJ, USA), and then embedded in paraffin. Paraffin blocks were cut into 5 μm thick sections, which were de-paraffinized and rehydrated after mounting on glass slides. Masson’s trichrome staining was performed using the DAKO Masson’s Trichrome Stain kit on the Artisan™ Link Special Stains Instrument (Dako North America, Inc., Agilent Technologies, Carpinteria, CA, USA). Collagen in the coronary wall stained blue and SMCs stained red. Collagen content measurements were performed within the entire medial layer and atheroma. Hematoxylin and eosin (H&E) staining was performed using the H&E Staining Kits from Tissue-Tek^®^ Prisma^®^ Automated Slide Stainers. Sections were examined using a Nikon microscope. Images were taken randomly from at least four different areas of the ring at 4× or 20× magnification. Image-Pro was used to quantify the positive staining. Results were expressed as a percentage of blue collagen staining over total area in the medial layer or the atheroma.

Immunostaining was performed using the following antibodies: the α-smooth muscle actin antibody (dilution 1:50, α-SMA, human, Sigma, St. Louis, MO, USA), the TRPC1 antibody (dilution 1:50, gift from Dr. Tsiokas), the TPRC6 antibody (dilution 1:25, S-20, Santa Cruz Biotechnology, Dallas, TX, USA), the CD163 antibody (1:100, Vector, Burlingham, CA, USA), the CD4 antibody (ready-to-use, IR649, DAKO, Glostrup, Denmark), and the CD33 antibody (ready-to-use, PWS44, Cell Marque, Rocklin, CA, USA). After incubations with the primary antibodies at room temperature, the sections were then probed using the DAKO Flex system (SMA, CD163, and CD4), the DAKO Omnis (CD33), the DAKO Envision + mouse (TRPC1) or with the secondary biotin AffiniPure donkey anti-goat antibody for TRPC6 (dilution factor 1:100, The Jackson ImmunoResearch Laboratory, West Grove, PA, USA), followed by the DAKO LSAB2 horseradish peroxidase-conjugated polymer. All washes were made using the DAKO wash buffer. The brown color was developed using diaminobenzidine. The staining density was quantified using the DAB analysis module of Image-Pro Premier 9.1 (Media Cybernetics Inc., Warrendale, PA, USA). The specific staining is reported after normalization to adventitia background staining density.

### In vivo gene delivery

A micro-infusion Bullfrog catheter [21] (Mercator Medsystems, San Leandro, CA, USA) was used to deliver the cDNA into the coronary artery wall and a custom made intravascular electroporation catheter [45] (Cardigant Medical Inc., Manhattan Beach, CA, USA) to transiently electropermeabilize SMCs, allowing their loading with cDNA. Since both catheters were designed for 3 mm arteries, before performing an in vivo gene delivery procedure, a coronary artery segment of 3 mm diameter was identified using IVUS. The segment chosen was a site that was close to a prominent coronary artery branch. This allowed identification of the segment following dissection. After completing the IVUS procedure, the IVUS catheter was withdrawn from the coronary artery and the micro-infusion Bullfrog catheter was advanced into the same artery under angiographic guidance to the selected artery segment. The 130 μm diameter injecting needle was deployed into the coronary vascular wall, and an injection of 0.7–1 ml of 1 mg/ml dominant-negative (DN) pig TRPC6 (L645A/F646A/W647A) cDNA in the pcDNA3.1 vector or the empty vector in sterile NaCl solution was performed. The needle was retracted and the injection catheter was withdrawn. The intravascular electroporation catheter was deployed to the site of injection and in vivo electroporation was performed using three square 20 ms voltage pulses of 75 V with an interval of 1 s. After the intra-coronary intervention was completed, the catheters and the guide wire were withdrawn and the incision was sutured. Aspirin (300 mg) was given to the pig by mouth with the feed a day before and a day after surgery. The expression of DN-TRPC6 was driven with the human cytomegalovirus immediate-early promoter of the pcDNA3.1 vector. The in vivo electroporated pigs were euthanized 48 h after the in vivo cDNA delivery procedure.

### Isometric tension measurement

Isolated coronary arteries were cut into 2–3 mm rings and mounted in the organ baths containing Krebs solution saturated with a gas mixture of 95% O_2_ and 5% CO_2_ at 37 °C. The optimal length was determined as described elsewhere [8, 11, 24]. This usually resulted in a preload value of ~3 g. Both KCl and histamine were added directly to the organ baths. The standard Krebs buffer contained (mM): 131.5 NaCl, 5 KCl, 2.5 CaCl_2_, 1.2 NaH2PO_4_, 1.2 MgCl_2_, 25 NaHCO_3_, and 10 glucose. Vessel tensions were measured using a GlobalTown Microtechnology (Sarasota, FL, USA) wire myograph. Active tension was determined as the difference of the peak and baseline tension.

### Front-surface fluorometry in coronary artery rings

A monochromator-equipped imaging system (TILL-Photonics, Martinsreid, Germany) was used to monitor intracellular Ca^2+^ changes in coronary artery rings. Cross-sectioned rings (~1 mm thick) were loaded with fura-2 by incubating with 8 µM fura-2AM (Molecular Probes, Inc., Eugene, OR, USA) for 6–10 h in a phosphate-buffered saline (DPBS, Cat # 14287, ThermoFisher Scientific, Waltham, MA, USA) solution containing 0.9 mM Ca^2+^, 0.49 Mg^2+^, supplemented with 5.55 mM d-glucose, 0.33 mM sodium pyruvate, and 2% bovine serum albumin (BSA), at room temperature in the dark. Rings were washed three times with PBS without fura-2AM and incubated in solutions without fluorescent dye for additional 30–60 min at room temperature. Fura-2 fluorescence was induced by alternating excitation at 345 and 380 nm. The obtained data were analyzed using TILLvisION software. The background fluorescence measured in a ring free area was subtracted from all fluorescence values before the determination of the F345/F380 ratio. The peak amplitudes of KCl and histamine were determined by subtracting the baseline ratio. The histamine steady state amplitude values were measured at the time point of 3.3 min after the histamine response peaked. The standard extracellular solution for fluorescence measurements contained (mM): 140 NaCl, 2.5 KCl, 1 MgCl_2_, 5 CaCl_2_, 4 glucose, 10 HEPES (pH 7.2).

### Preparation of porcine peripheral blood mononuclear cells (PBMCs) and enrichment of monocytes

Porcine blood was collected in sodium heparin-containing tubes and immediately used for PBMC isolation with Ficoll-Paque PLUS (GE Healthcare Bio-Sciences, PA, USA) according to instructions by the manufacturer. 10 million PBMCs cells were stained with PerCPCy5.5 Anti-Pig CD3ε (12 mg/ml, clone: BB23-8E6-8c8, BD Pharmingen) and R-phycoerythrin Anti-Pig CD79a (8 mg/ml, clone: HM47, eBioscience) antibodies and then sorted on the BD SORP Aria instrument. The stained PBMCs were first gated with high Forward Scatter (FSC-A) and high Side-Scatter (SSC-A) of light to collect monocyte subpopulations, which were further sorted to exclude the cells positive for CD3ε and CD79a. Enrichment was assessed by staining the initial PBMC samples and sorted monocyte samples with fluorescein isothiocyanate anti-pig monocyte/granulocytes CD172a (10 mg/ml, clone 74-2215A, BD Pharmingen) and PerCPCy5.5 anti-pig CD3ε antibodies. The monocyte enrichment was 77–82% (*n* = 4).

### Coronary artery organ-culture

Lean pig coronary arteries (3 mm long strips or rings) were pretreated for 2–7 days with either vehicle (DMSO), aldosterone (100 nM), a mixture of aldosterone and spironolactone (100 nM and 10 μM, respectively), or spironolactone (10 μM) in EMEM (cat # 30-2003, ATCC, Manassas, VA, USA) supplemented with 2% bovine serum albumin, 50 units penicillin, and 50 μg/ml streptomycin. The medium was replaced daily. Organ-cultured coronary artery segments were maintained at 37 °C in a water-jacketed 5% CO_2_ incubator.

### Monocyte adhesion assay

Organ-cultured coronary artery strips were treated with vehicle (DMSO), aldosterone (100 nM), a mixture of aldosterone and spironolactone (100 nM and 10 μM, respectively), or spironolactone (10 μM) for 48 h. The isolated pig peripheral blood monocytes were loaded with Carboxy SNARF-1 (10 μM, Spiro[7H-benzo [c]xanthene-7,1′(3H)-isobenzofuran]-ar′-carboxylic acid, 3-(acetyloxy)-10-(dimethylamino)-3′-oxo-, (acetyloxy)methyl ester; ThermoFisher Scientific) for 30 min at room temperature. The SNARF-1 loaded monocytes (3 × 10^4^–2 × 10^5^ cells per well) were added to the four listed above groups of treated organ-cultured strips and incubated for 2 h at 37 °C on the stage of a horizontal New Brunswick Scientific Shaker, rotating at 75 rpm in the atmosphere of 5% CO_2_ and 95% oxygen. After the incubation period, the strips were gently washed three times with PBS supplemented with Ca^2+^, Mg^2+^, glucose, and pyruvate (catalog # 14287, Thermo Fisher Scientific) and then were fixed in 10% phosphate-buffered formalin. The sections were next mounted on glass slides with the lumen facing up using Vectashield Antifade Mounting Medium with 4′,6-diamidino-2-phenylindole (Catalog #: H-1200, Vector Laboratories, Inc., Burlingame, CA, USA). The fluorescence of SNARF-1 was excited at 570 nm and the emitted light was collected with a longpass filter of 600 nm, and the fluorescence of 4′,6-diamidino-2-phenylindole (DAPI) was excited at 360 nm and the emitted light was collected with a band filter of 400–450 nm. Blue DAPI fluorescence was used to focus on the endothelium layer of the coronary strips, and then the red fluorescence of SNARF was used to identify the attached monocytes. The number of adherent monocytes was determined using the Till Photonics single-cell fluorescence imaging system by counting the red fluorescing cells attached to the surface of the endothelium. Each value represents an average of the adherent monocyte counts observed in three different images for each strip.

### Pig macrophage culture and differentiation

Freshly isolated peripheral blood mononuclear cells were suspended in the macrophage growth medium and were then plated on plastic 24-well plates (BD Biosciences, Thermo Fisher Scientific). The macrophage growth medium consisted of RPMI-1640 (Gibco, Thermo Fisher Scientific), supplemented with 15% L929 conditioned medium, 15% horse serum, 5% FBS, 1 mM glutamine, 50 units penicillin, and 50 μg/ml streptomycin. Cells were cultured for 3 days, and on day 3, the old medium and floating cells were removed from the wells. The attached cells were washed one time with PBS and then with fresh macrophage growth medium and cultured further for an additional 2 days. On day 5, the old macrophage growth medium was removed leaving 0.5 ml in each well, and then fresh macrophage growth medium was added to the wells. On day 7, the differentiated macrophages were used for total mRNA isolation as described later. The L929 conditional medium was prepared as follows. L929 cells (ATCC) were grown in complete DMEM supplemented with 10% fetal bovine serum, 50 units penicillin, and 50 μg/ml streptomycin until 90% confluence for four days. The supernatant was collected in 50 ml centrifuge tubes, centrifuged at 10,000* g* to remove cell debris and after filtration (20 μm) used as the conditioned L929 medium.

### Real-time quantitative PCR (RT-qPCR)

Total RNA from snap-frozen Lean pig coronary artery rings organ-cultured for 36 h was isolated using the Trizol method (Invitrogen) with a subsequent deoxyribonuclease treatment step to eliminate any possible traces of the genomic DNA in the total RNA. The Bio-Rad iScript cDNA synthesis kit was utilized to reverse-transcribe cDNA using total RNAs as templates. Total RNA from macrophage cultures was isolated using the RNeasy Mini kit (Qiagen) according to manufacturer’s instructions. The superscript VILO cDNA synthesis kit was used to reverse-transcribe cDNA using total RNAs as templates. The Applied Biosystems (Foster City, CA, USA) 7500 Real-Time PCR System was utilized to perform RT-qPCR. The data were quantified using the standard ΔΔCt method. The endogenous controls (18S ribosomal (r) RNA or β_2_ micro globulin) were amplified with TaqMan Universal PCR Master Mix (Applied Biosystems), whereas TRPC1 and TRPC6 were amplified using SYBR Green Master mix (Applied Biosystems). The sequences of primer sets were as follows: 5′-TAGCAACCAGCCCCAGTCAGTCT-3′ (TRPC6, forward), 5′-AGGCCGTTCAATCCGAGCAC-3′ (TRPC6, reverse), 5′-CGATGCTCTTAGCTGAGTGT-3′ (18S rRNA, forward), and 5′-GGTCCAAGAATTTCACCTCT-3′ (18S rRNA, reverse). The RT^2^ qPCR primer assays for TRPC1 and β_2_ micro globulin were from QIAGEN (Mansfield, MA, USA; Cat.#: PPS03885A and Cat.# PPS00376A, respectively). The RT^2^ qPCR primer sequences were not disclosed by the manufacture. The no-reverse-transcriptase control was performed and was negative.

### Chemicals

All inorganic salts and buffers, histamine, and α-smooth muscle actin antibodies were purchased from Sigma (St. Louis, MO, USA). The TRPC6 antibody was from Santa Cruz (Dallas, TX, USA). Fura-2AM, Vectashield Antifade Mounting Medium, and Carboxy SNARF-1 were from Thermo Fisher Scientific.

### Statistical analysis

All data are presented as mean ± standard error of the mean (SEM). The unpaired *t* test was used to determine whether there is a statistically significant difference between the two data sets with normally distributed populations and equal variances. The one-way ANOVA test followed by the post hoc all pairwise multiple comparison Student–Newman–Keuls test was used to determine whether there was a significant difference between the means of multiple experimental groups with normally distributed populations and equal variances. The Kruskal–Wallis analysis of variance on ranks test followed by the post hoc all pairwise multiple comparisons Dunn’s test was used to compare the data sets with non-normally distributed populations and unequal variances. The two-way ANOVA test followed by the Student–Newman–Keuls post hoc all pairwise multiple comparison test was used to compare the experimental groups affected by two different factors when the data sets were normally distributed populations with equal variances. The SigmaPlot 13 software package (Systat Software Inc., San Jose, CA, USA) was used to perform the statistical analyses. The results were considered significantly different if the *p* value was <0.05.

## Results

To investigate the effect of long-term spironolactone treatment on TRPC expression and coronary pathology in metabolic syndrome pigs, Ossabaw miniature pigs were subdivided into three groups: Lean, metabolic syndrome (MetS), and metabolic syndrome treated with spironolactone (MetS-SN). All of the pigs were initially raised on the standard chow to an age of about 6–8 months. The pigs were then fed diets according to their group for an additional 7–9 months. The diets were as follows: the same standard chow (Lean group), an atherogenic diet (1 kg/day, MetS group), or the atherogenic diet supplemented with spironolactone (3 mg/kg body weight/day, MetS-SN group).

### Metabolic phenotype

**Fig. 1:**
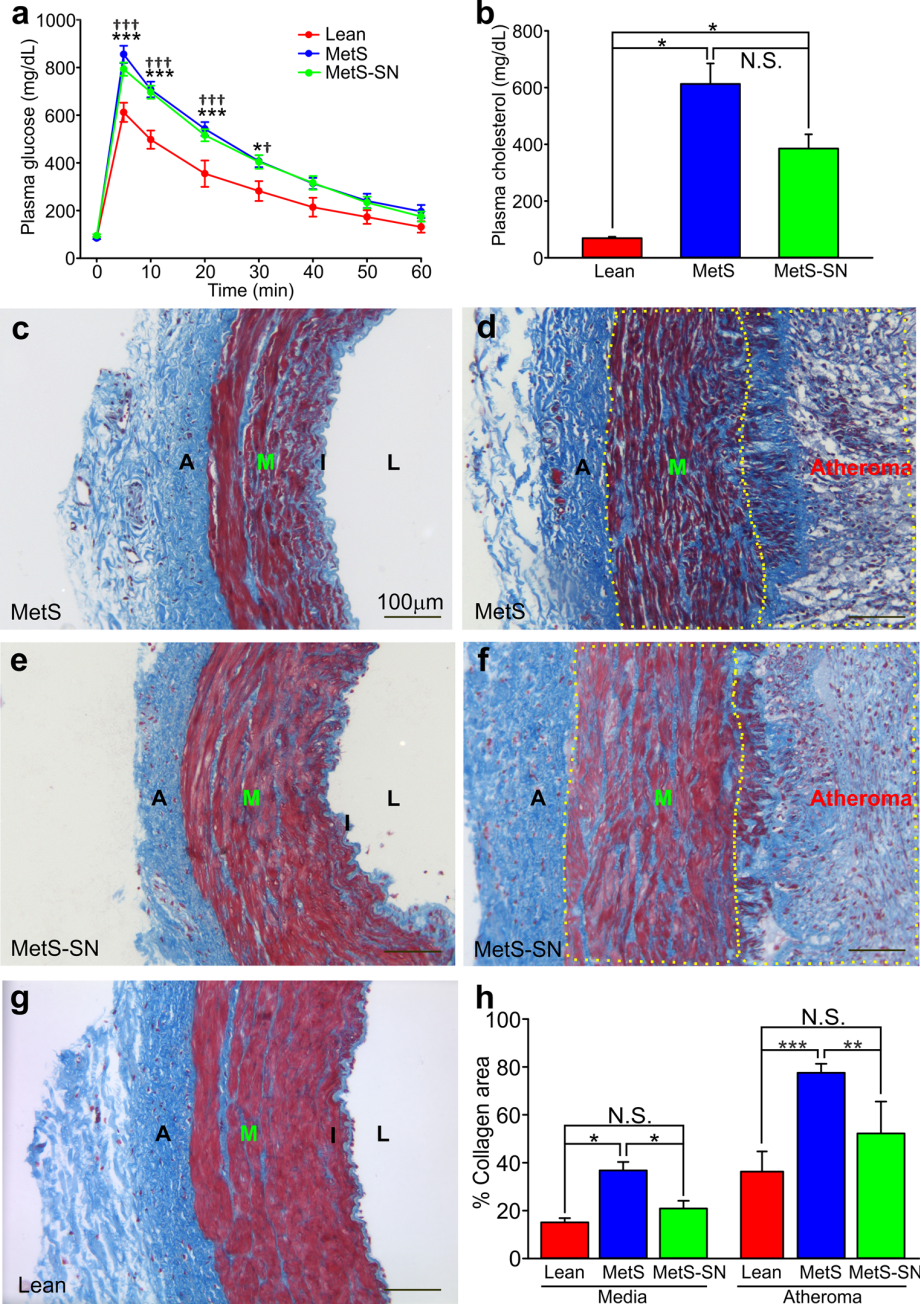
Metabolic phenotype and coronary wall collagen content in Lean, MetS, and MetS-SN pigs. **a**, Time course of changes in blood glucose concentration obtained during intravascular glucose tolerance test. MetS or MetS-SN pigs IVGTTs are compared to that in Lean pigs (*n* = 5–8 pigs at each data point). *P < 0.05 (MetS vs. Lean); ^†^ *P* < 0.05 (MetS-SN vs. Lean); ***P < 0.001; ^†††^ *P* < 0.001; the two-way ANOVA test followed by the post hoc Student–Newman–Keuls test. **b** Summary for mean total plasma cholesterol concentrations determined in samples from the three experimental groups. *P < 0.05; Kruskal–Wallis one-way analysis of variance on ranks followed by the Dunn’s post hoc test; **c**–**g** Masson’s trichrome staining of coronary artery wall sections. Both non-atherosclerotic coronary artery sections (**c**, **e**) and atherosclerotic coronary artery sections (**d**, **f**) are shown in the MetS and MetS-SN groups. Collagen is stained *blue* and smooth muscles are stained *red*. *Bars* 100 μm. *A* adventitia, *M* medial layer, *I* intima, *L* lumen, magnification ×20. **h** Quantification of collagen percent area in the media layer and the atherosclerotic lesions. *P < 0.05; **P < 0.01; ***P < 0.001; ^†^ *P* < 0.05; *NS* non-significant. The two-way aNOVA test followed by the Student–Newman–Keuls post hoc test

The pigs from the MetS and MetS-SN groups were obese as compared to the Lean group with significantly greater body weight (Table 1). However, there was no significant difference between the average body weights in the MetS and MetS-SN groups. MetS pigs exhibited elevated mean systolic blood pressure, but it was not significantly different from that observed in the MetS-SN group. The values of mean diastolic blood pressure, serum sodium, serum potassium, and serum triglycerides were not significantly different among the tested groups (Table 1). Pigs from the MetS and MetS-SN groups presented with impaired glucose tolerance when compared to Lean pigs (Fig. 1a). Blood glucose levels were higher in MetS and MetS-SN pigs when compared with Lean pigs at the 5, 10, 20, and 30 min time points following a bolus of intravenous glucose of 1 g/kg of body weight (Fig. 1a). The mean total plasma cholesterol concentration was markedly elevated in the MetS and MetS-SN groups as compared to that observed in the Lean group (Lean: 69 ± 5 mg/dl, *n* = 12; MetS: 613 ± 72 mg/dl, *n* = 10; MetS-SN: 385 ± 50 mg/dl, *n* = 9; Fig. 1b). There was no difference between the total plasma cholesterol concentrations observed in the MetS and MetS-SN groups, indicating that spironolactone treatment did not significantly affect the total plasma cholesterol levels in the pigs.

**Table 1:** Phenotypic characteristics of pig groups

	Lean	MetS	MetS-SN	*P* values		
				L vs. MetS	L vs. MetS-SN	MetS vs. MetS-SN
Number of animals	7	7	6			
Body weight (kg)	49 ± 4	84 ± 3	79 ± 3	<0.001	<0.001	0.3
SBP (mmHg)	131 ± 3	151 ± 3	138 ± 7	0.01	0.27	0.06
DBP (mmHg)	75 ± 4	84 ± 4	71 ± 4	NS	NS	NS
Triglycerides (mg/dl)	28.9 ± 6.3	41.7 ± 13.0	30.7 ± 3.3	NS	NS	NS
Sodium (mEq/l)	136.3 ± 1.0	139.4 ± 0.7	138.2 ± 1.2	NS	NS	NS
Potassium (mEq/l)	4.9 ± 0.7	4.4 ± 0.3	4.3 ± 0.2	NS	NS	NS

The one-way ANOVA test followed by the Student–Newman–Keuls post hoc test

*L* lean, *MetS* metabolic syndrome, *MetS-SN* metabolic syndrome group treated with spironolactone, *SBP and DBP* systolic and diastolic blood pressure, *NS* no significant different

### Coronary artery disease

Collagen content is an important characteristic of atherosclerotic plaques, as increased collagen deposits are linked to coronary stenosis and increased plaque stability. Therefore, we investigated whether collagen deposition in the coronary artery medial layer and atheroma varied among the experimental groups. The Masson’s trichrome staining showed that collagen content was significantly higher in the medial layer of MetS pigs (36.8 ± 3.5%, *n* = 10) compared to Lean pigs (15.1 ± 1.8%, *n* = 6; Fig. 1c–h; Supp. Figure 1). Long-term spironolactone treatments significantly decreased total collagen deposition in the medial layer (20.9 ± 3.2%, *n* = 6; MetS-SN group). Atheroma collagen content was significantly decreased in MetS-SN pigs compared to that in MetS pigs (Lean atheroma 29.9 ± 10.3%, *n* = 3; MetS-SN atheroma 52.2 ± 13.3%, *n* = 4; MetS atheroma 77.5 ± 3.8%, *n* = 6; Fig. 1h).

**Fig. 2:**
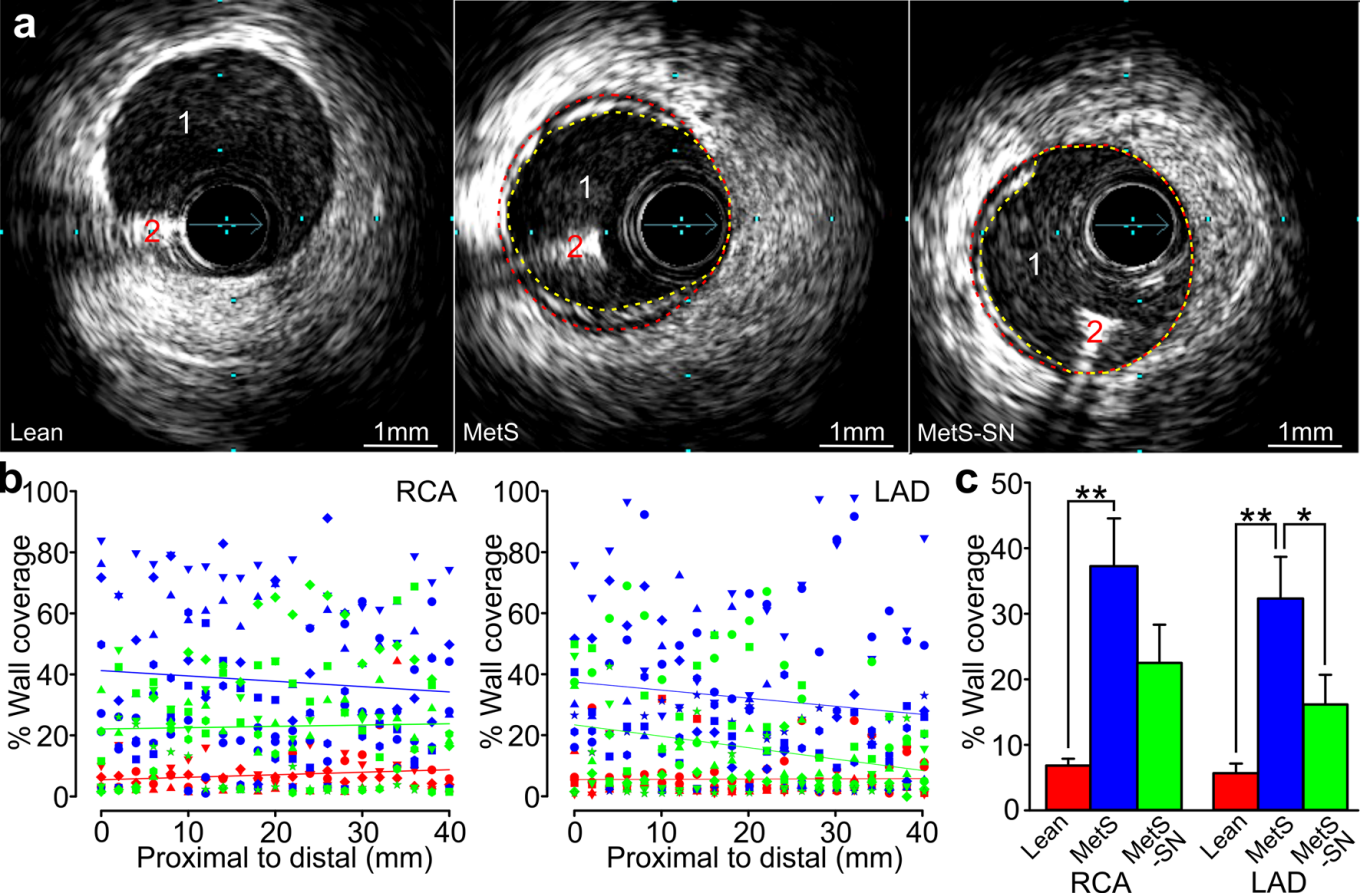
Intravascular ultrasound (IVUS) assessment of atherosclerosis in Lean, MetS, and MetS-SN pigs. **a** Sample IVUS cross-sectional images of coronary arteries are shown. The external elastic membrane border and actual lumen border are indicated with *red* and *yellow lines,* respectively. *1* Coronary artery lumen. *2* The angioplasty guidewire artifact. **b** Scatter plots of the percent wall coverage along the RCA and LAD of each pig of three groups coded with different *symbols* and *colors* (Lean = *red*; MetS = *blue*; and MetS-SN = *green*) determined using IVUS. **c** Comparison of average wall coverage determined using IVUS in Lean (*red*), MetS (*blue*) and MetS-SN (*green*) groups. *P < 0.05; **P < 0.01. The two-way ANOVA test followed by the Student–Newman–Keuls post hoc test

**Fig. 3:**
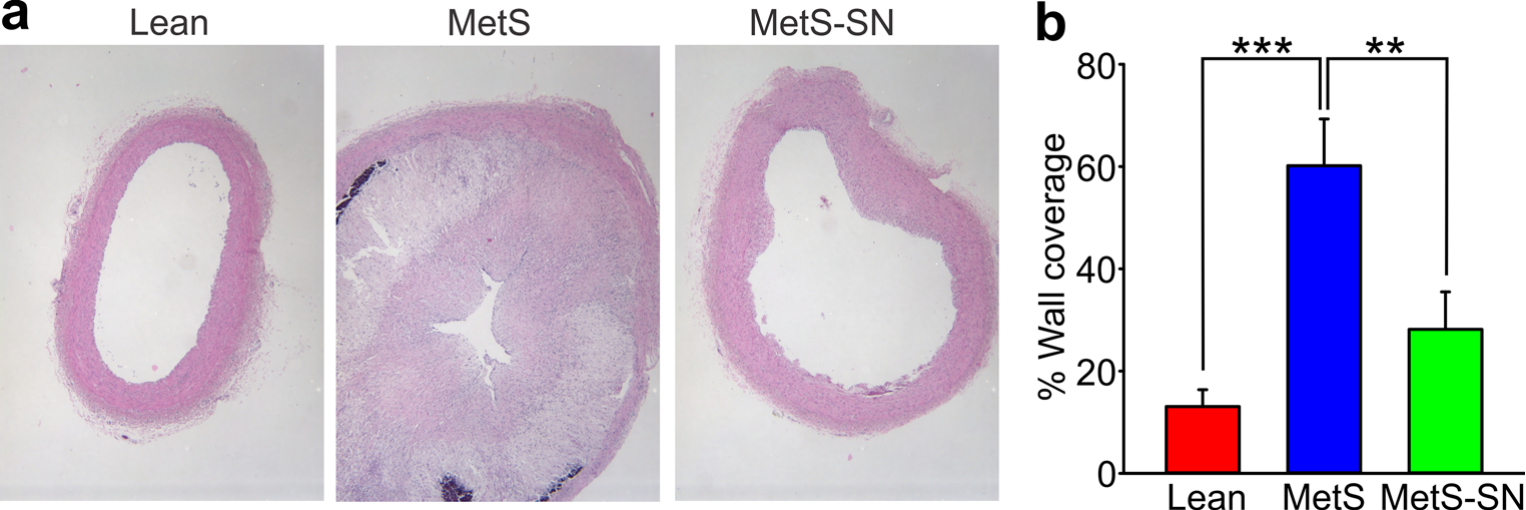
Atherosclerosis assessment using histology. **a**, Sample images of H&E staining of coronary artery sections from the three tested groups. **b** Summary of histological evaluation of atheroma percent wall coverage in cross sections of RCAs from Lean, MetS and MetS-SN groups. **P < 0.01; ***P < 0.001. The one-way ANOVA test followed by the Student–Newman–Keuls post hoc test

To obtain an initial assessment of the extent of atherosclerosis in coronary arteries of Lean, MetS, and MetS-SN groups, we first performed IVUS measurements (Fig. 2). Lean coronary arteries presented only minimal atherosclerosis (Fig. 2a, Lean). Conversely, MetS coronary arteries exhibited increased atherosclerosis (Fig. 2a). The percent wall coverage was significantly higher in coronary arteries from left anterior descending (LAD) and right coronary arteries (RCA) in MetS pigs compared to Lean pigs (RCA: Lean 6.8 ± 1.1%, *n* = 4, MetS 37.4 ± 7.2%, *n* = 7; LAD: Lean 5.7 ± 1.5%, *n* = 5, MetS 32.3 ± 6.4%, *n* = 7; Fig. 2b, c). Long-term spironolactone treatment significantly reduced atherosclerosis in the LAD MetS-SN group (the percent wall coverage: 16.1 ± 4.5% for Met-SN LAD, *n* = 6, and 22.5 ± 5.8% for MetS-SN RCA, *n* = 6; Fig. 2c). However, likely due to the limited number of pigs examined with IVUS no significant difference was detected between the RCA-MetS and Met-SN groups. However, histology to assess the degree of atherosclerosis was performed on all pigs including those MetS pigs that died during the study. The mean percent wall coverage was significantly increased in MetS RCAs as compared to that in Lean RCAs and significantly reduced in MetS-SN RCAs as compared to the mean value observed in pigs from the MetS group (RCA-MetS: 60.2 ± 9%, *n* = 13; RCA-Lean: 13.1 ± 3.3%, *n* = 9; and RCA-MetS-SN: 28.2 ± 7.3%, *n* = 9; Fig. 3). These data indicate that long-term spironolactone treatment decreases MetS-associated collagen deposition and the degree of atherosclerosis.

### MetS coronary arteries exhibit increased histamine-induced contractions and intracellular Ca^2+^ transients

**Fig. 4:**
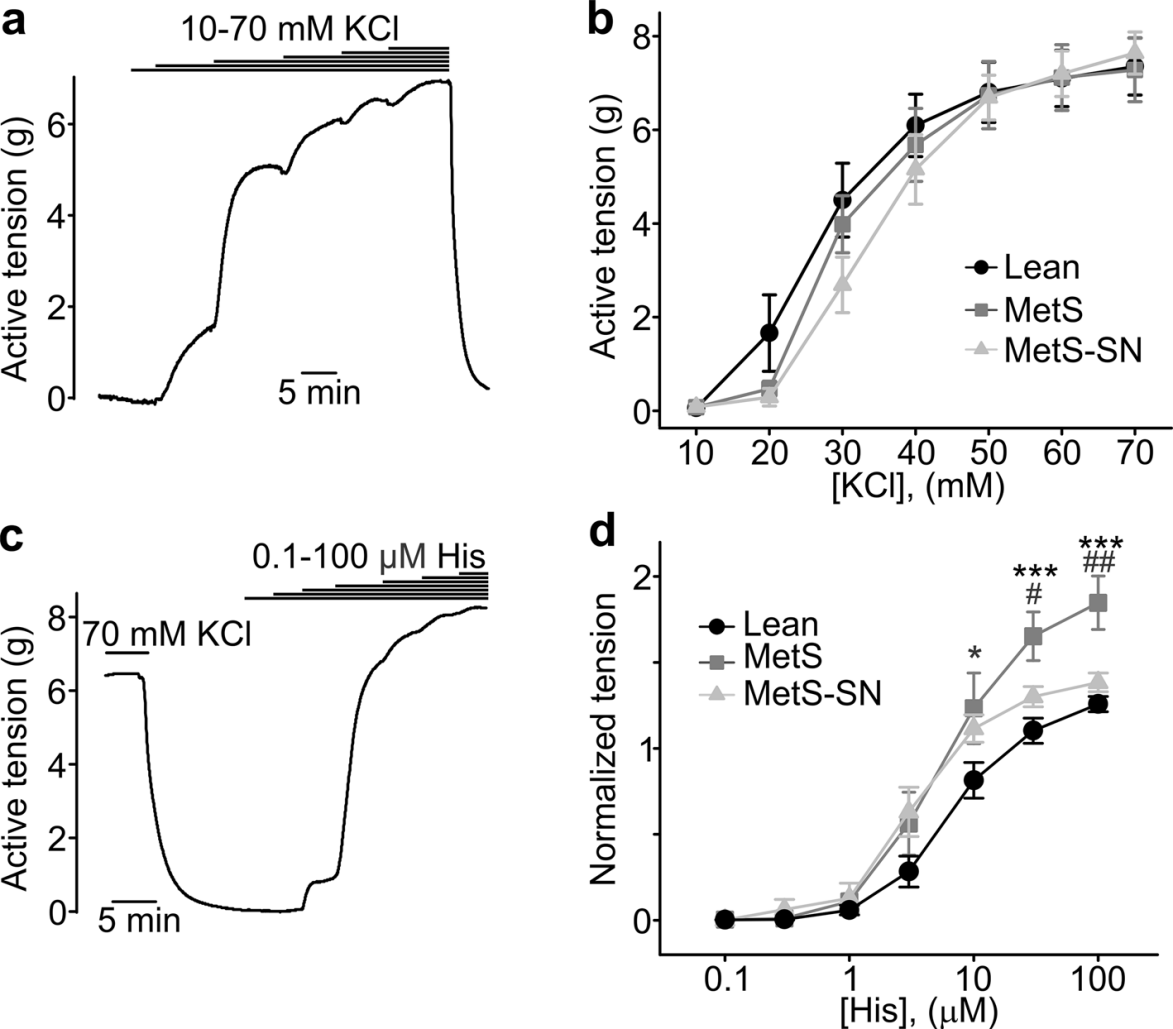
Isometric tension measurements. **a**, **c** Sample traces of active tension changes induced by KCl or histamine in coronary artery rings from the Lean group are shown. Contractions were induced by cumulatively increasing concentrations of KCl (10–70 mM, **a**) or histamine (10^−7^ to 10^−4^ M, **c**). **b**, **d** Concentration–response curves for KCl and histamine. *His* histamine. The mean active tension value for each data point was calculated by averaging the mean active tension values obtained from 2 to 4 rings in each pig. *P < 0.05; ***P < 0.001; for MetS vs. Lean and ^#^ *P* < 0.05; ^##^ *P* < 0.01 for MetS vs. MetS-SN. The two-way ANOVA test followed by the Student–Newman–Keuls post hoc test

Isometric tension measurements were performed to assess the ex vivo contractility of coronary arteries from the three tested groups. Coronary artery rings across the groups exhibited similar concentration-dependent contraction stimulated by KCl-mediated depolarization of smooth muscles (Fig. 4a, b). The EC_50_ values were as follows: Lean: 28 ± 2 mM; MetS: 29 ± 2 mM; MetS-SN: 33 ± 2 mM. Histamine (10^−7^ to 10^−4^ M) also induced concentration-dependent contractions of coronary artery rings from all of the three tested groups (Fig. 4c, d); and contractions induced by 10, 30, and 100 µM histamine were significantly increased in coronary arteries from MetS pigs as compared to those from Lean and MetS-SN pigs (for 10 µM—Lean: 1.05 ± 0.05, *n* = 13 vs. MetS 1.23 ± 0.1, *n* = 7; for 30 µM—Lean: 1.10 ± 0.07, *n* = 13 vs. MetS: 1.65 ± 0.14, *n* = 7; MetS vs. MetS-SN: 1.3 ± 0.06, *n* = 9; for 100 µM—Lean: 1.26 ± 0.04, *n* = 13 vs. MetS: 1.85 ± 0.15, *n* = 7; MetS vs. MetS-SN: 1.38 ± 0.06, *n* = 9). However, the EC_50_ values for histamine (Lean 6.9 ± 1.1 µM; MetS 3.5 ± 0.6 µM; MetS-SN 5.9 ± 1.5 µM; Fig. 4d) were not significantly different among the tested groups.

**Fig. 5:**
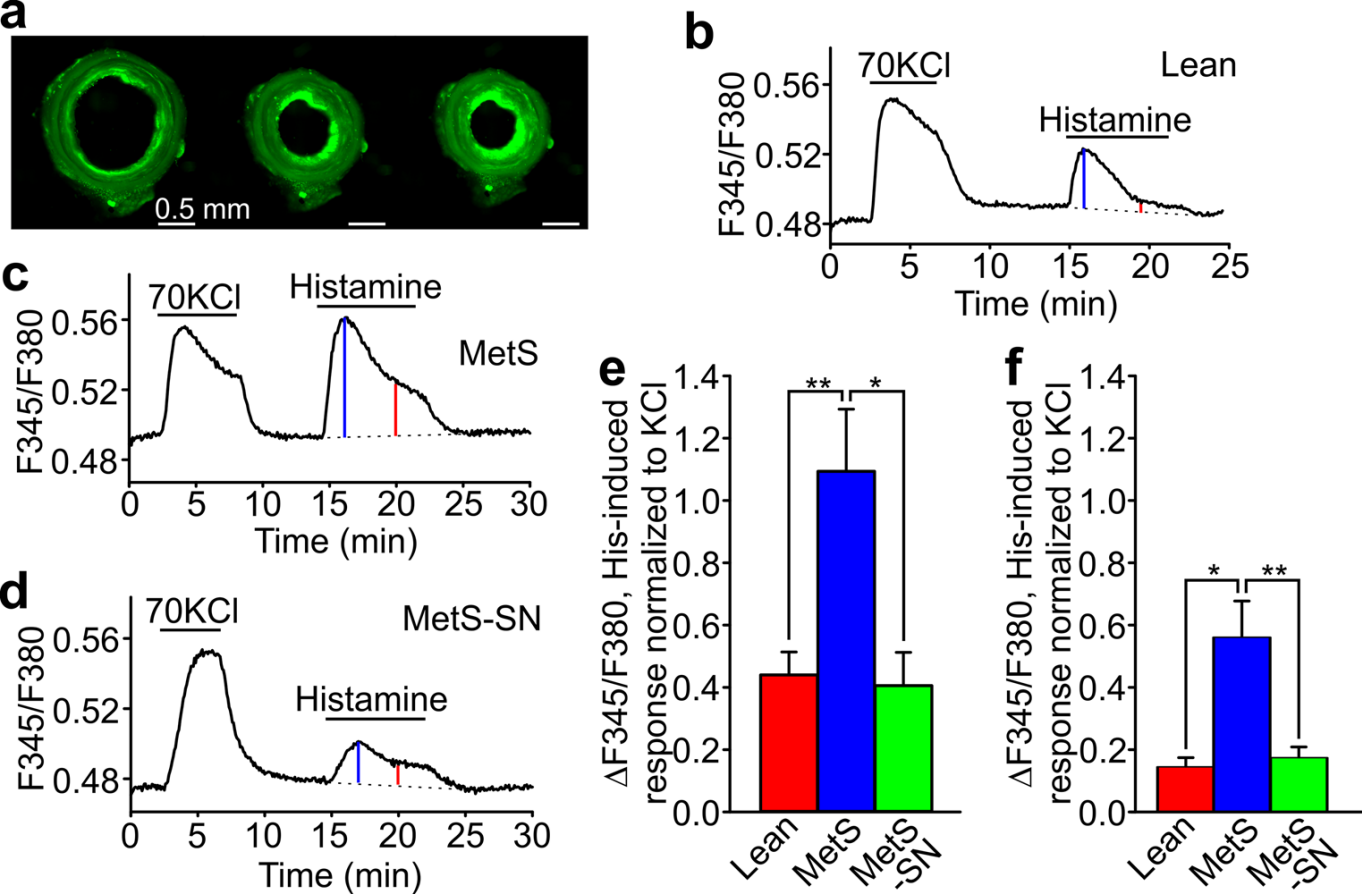
Front-surface fluorometry in coronary artery rings loaded with Fura-2. **a** Fluorescence images of a coronary artery ring from a Lean pig, which were obtained at an excitation wavelength of 380 nM (*left panel* control; *middle panel* under 70 mM KCl; *right panel* under 40 μM histamine). **b**–**d** Sample traces of [Ca^2+^]_i_ changes in coronary artery rings from Lean (**a**), MetS (**b**), and MetS-SN (**c**) rings are shown. KCl and histamine (40 μM) were added at the times indicated with the *horizontal bars*. **e** Summary of data for the peak (*blue vertical line* in **b**–**d**) values of [Ca^2+^]_i_ responses to histamine (40 μM) normalized to the peak of KCl response in the tested rings. *P < 0.05; **P < 0.01. The one-way ANOVA test followed by the Student–Newman–Keuls post hoc test. **f** Steady state phase (*red vertical line* in **b**–**d**) values of [Ca^2+^]_i_ responses to histamine (40 μM) normalized to the peak of KCl response in the tested rings. The steady state value was calculated at 3.3 min after the peak value of the histamine response was reached. Kruskal–Wallis one-way analysis of variance on ranks followed by the Dunn’s post hoc test. *P < 0.05; **P < 0.01. *His* histamine

We also employed front-surface fluorometry to assess intracellular Ca^2+^ ([Ca^2+^]_i_) changes in response to KCl and histamine in fura-2 loaded rings (Fig. 5a). The peak amplitudes of 70 mM KCl-stimulated Ca^2+^ influx in Lean, MetS, and MetS-SN groups did not differ significantly (Lean 0.10 ± 0.02, *n* = 7; MetS 0.06 ± 0.01, *n* = 9; MetS-SN 0.08 ± 0.02, *n* = 6). Conversely, the peak amplitudes of 40 µM histamine-induced Ca^2+^ influx were significantly larger in MetS coronary artery rings (1.1 ± 0.2, *n* = 9) than in Lean (0.4 ± 0.07, *n* = 7), and MetS-SN (0.4 ± 0.1, *n* = 6; Fig. 5b–f). The mean steady state values of histamine-induced [Ca^2+^]_i_ increases were greater in MetS rings (0.56 ± 0.10, *n* = 6) than in Lean rings (0.15 ± 0.03, *n* = 4) and MetS-SN rings (0.18 ± 0.03, *n* = 5; Fig. 5b–f). These data indicate that besides reducing atheroma formation, long-term spironolactone treatments attenuate histamine-induced [Ca^2+^]_i_ changes and vasoconstriction that is associated with MetS.

### MetS-associated coronary artery endothelial dysfunction can be prevented by long-term treatment with spironolactone

**Fig. 6:**
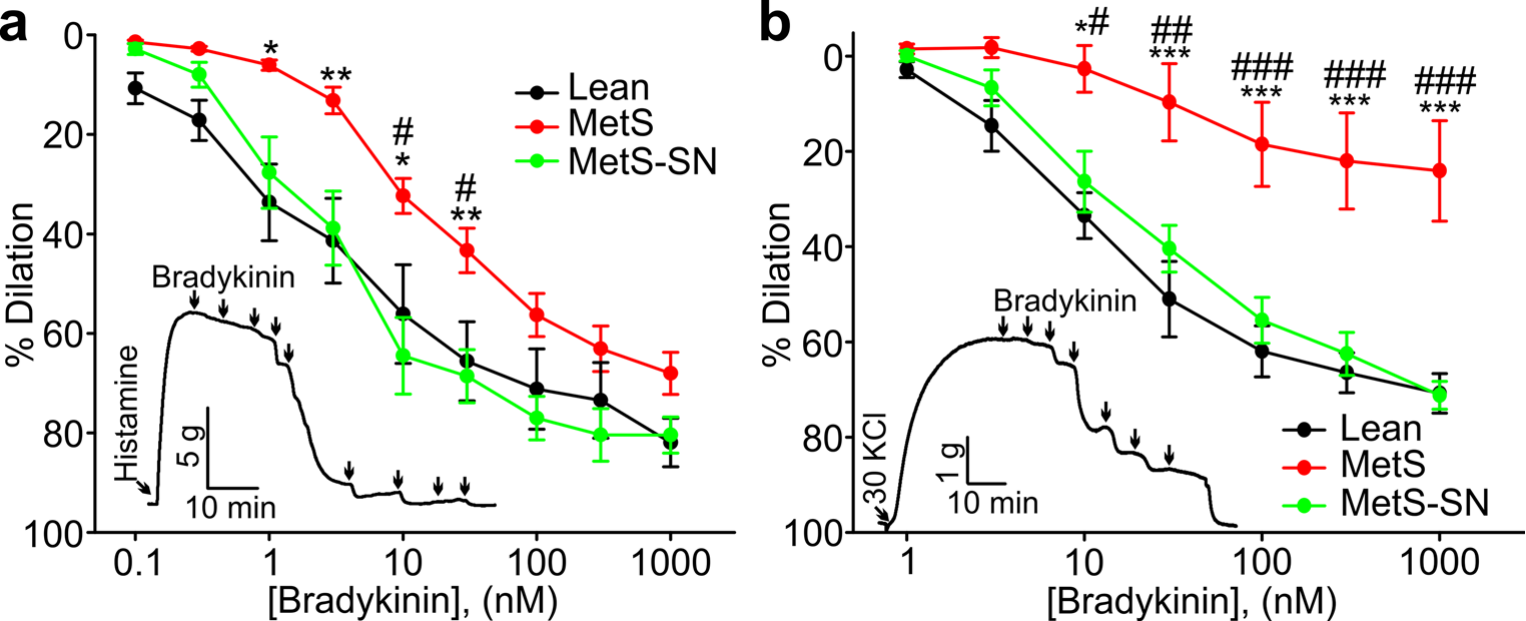
Assessment of endothelial function in coronary arteries from the three tested groups using isometric tension measurements. **a**, **b** Concentration–response curves for bradykinin-induced dilation in histamine- (**a**) and 30 mM KCl- (**b**) pre-contract coronary artery rings from Lean, MetS and MetS-SN pigs. Sample traces of bradykinin-induced coronary ring dilations are shown in *insets*. *P < 0.05, **P < 0.01, ***P < 0.001, *MetS vs. Lean; ^#^ *P* < 0.05, ^##^ *P* < 0.01, ^###^ *P* < 0.001, ^#^MetS vs. MetS-SN. The two-way ANOVA test followed by the Student–Newman–Keuls post hoc test

MetS coronary arteries are known to exhibit endothelial dysfunction. Indeed, Fig. 6a shows that concentration-dependent bradykinin-induced dilations were significantly decreased in MetS pig coronary arteries pre-contracted with 100 μM histamine (*n* = 10) as compared to those from Lean pigs (*n* = 7). Long-term treatment with spironolactone significantly improved endothelial function in MetS pig coronary arteries pre-contracted with 100 μM histamine (*n* = 6, Fig. 6a). Figure 6b shows that similar results were obtained in 30 mM KCl pre-contracted coronary artery rings (MetS, *n* = 5; Lean, *n* = 8, and MetS, *n* = 5). MetS coronary artery rings presented with markedly impaired bradykinin-induced dilations as compared to 30 mM KCl pre-contracted Lean pig coronary artery rings, and spironolactone-treated MetS-SN pigs showed significantly improved bradykinin-induced dilations.

### Pattern of TRPC immunostaining in the coronary artery wall

**Fig. 7:**
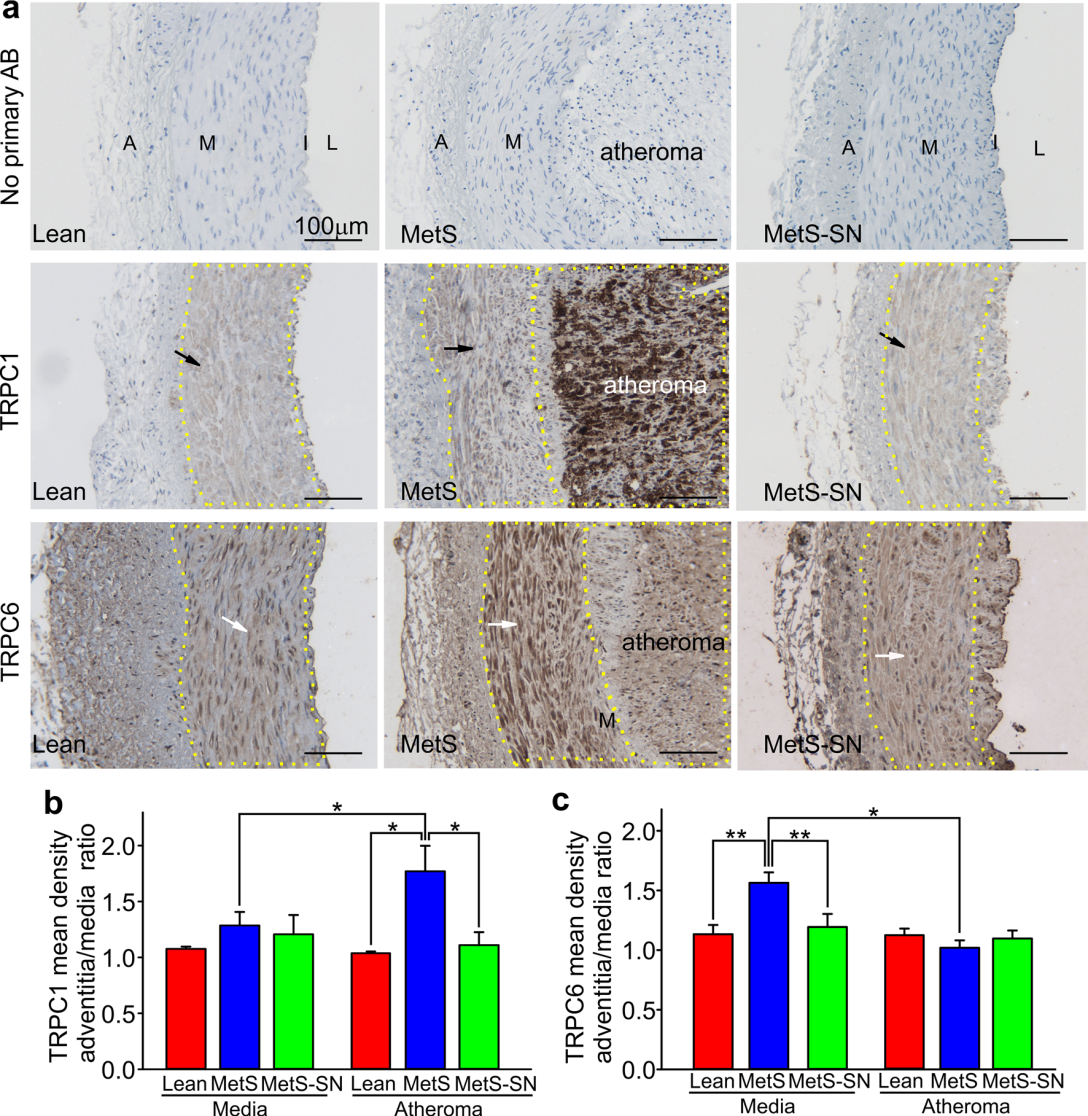
Assessment of TRPC expression pattern in the coronary artery wall. **a** Sample images of immunohistochemical staining of coronary artery ring sections from the tested groups. The *upper row* of images shows controls in which no primary antibody (AB) was added. The *middle row* of images show the sections stained with the TRPC1 antibody, whereas the *lower row* of images shows the sections stained with the TRPC6 antibodies. The *medial layer* is indicated with *black arrows* in TRPC1 images and *white arrows* in the TRPC6 images. The *dotted lines* show the borders between the coronary wall layers. *Scale bars* 100 µm. *A* adventitia, *M* media, *I* intima, *L* lumen. **b**, **c** Comparison of mean normalized density of immunostaining in the tested groups for TRPC1 and TRPC6, respectively. *P < 0.05; **P < 0.01. The two-way ANOVA test followed by the Student–Newman–Keuls post hoc test

To determine whether the MetS-associated increases in TRPC1 and TRPC6 protein expression occur locally or globally within the coronary wall immunohistochemical studies were performed. TRPC1 immunostaining was weak in the medial layer of all tested groups (Fig. 7a). The mean immunostaining density ratios of adventitia over media were as follows: 1.08 ± 0.03 (*n* = 4), 1.10 ± 0.02 (*n* = 9) and 1.06 ± 0.02 (*n* = 5), for Lean, MetS, and MetS-SN groups, respectively (Fig. 7b). Conversely, TRPC1 immunostaining was significantly greater in MetS atheromas than in MetS medial layer (MetS atheroma: 1.77 ± 0.23, *n* = 7). Spironolactone treatment significantly decreased TRPC1 immunostaining in the atheromas (MetS-SN atheroma 1.11 ± 0.12, *n* = 5, Fig. 7b). These data indicate that in Lean pigs, TRPC1 expression is low in the coronary artery wall with a particular localization to the medial layer. The channel’s protein expression is not significantly increased in the medial layer of MetS pigs. However, TRPC1 is highly expressed in the atheromas of MetS pigs.

In the coronary artery wall, TRPC6 immunostaining was localized to the medial layer in all the tested groups. Its density was similarly low in both adventitia and atheroma of MetS and MetS-SN pigs (MetS: 1.02 ± 0.06, *n* = 6; MetS-SN: 1.10 ± 0.07, *n* = 4, Fig. 7c). But, TRPC6 immunostaining density was much stronger in the medial layer of MetS pigs as compared to that in the Lean and MetS-SN medial layers, with the TRPC6 staining ratios of adventitia/medial being as follows: Lean 1.13 ± 0.08 (*n* = 6) vs. MetS 1.56 ± 0.09 (*n* = 9), and MetS vs. MetS-SN 1.19 ± 0.11 (*n* = 6). These data indicate that TRPC6 expression is increased in the medial layer, but not in atheromas of MetS coronary artery wall; and that TRPC6 upregulation in the MetS medial layer is diminished by spironolactone treatment (Suppl. Figure 2).

### Increased TRPC expression correlates with coronary artery pathology

**Fig. 8:**
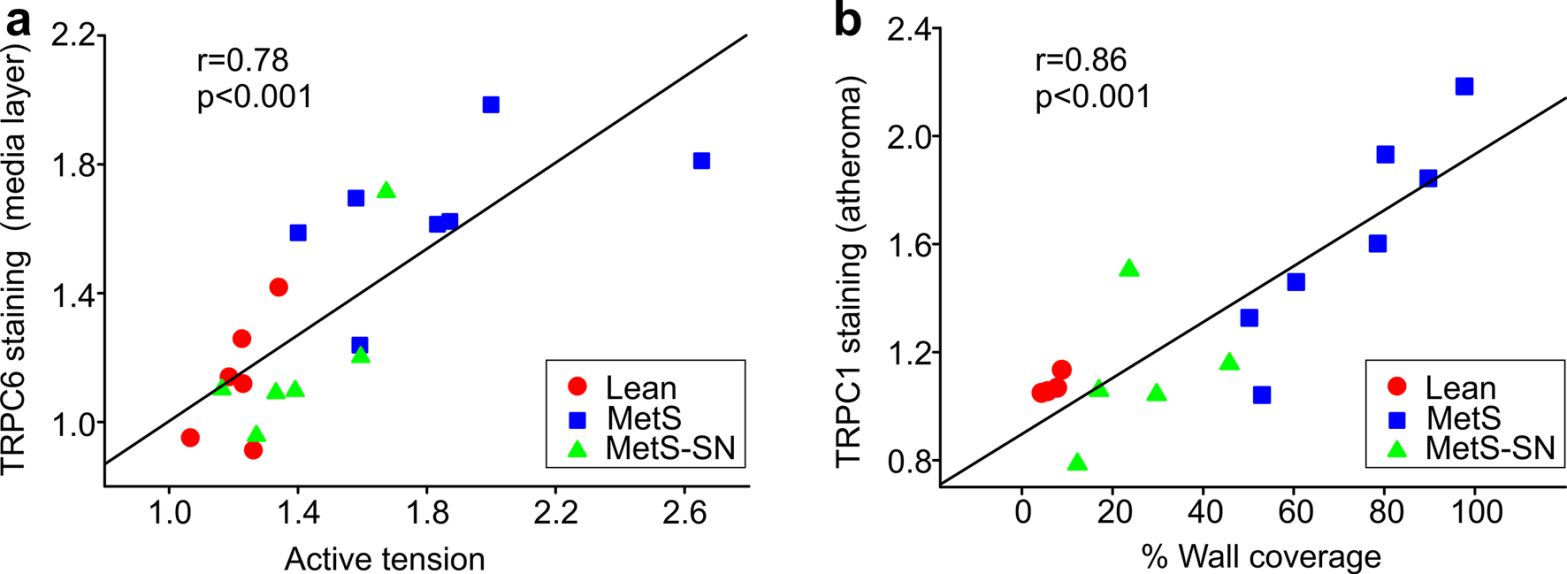
Pearson correlation analysis. **a**, **b** The correlation plots show strong correlations between: **a** TRPC6 expression and active tension induced by 100 μM His normalized to 70 mM KCl as shown in Figs. 4 and 7; and **b** TRPC1 expression and percentage wall coverage from Figs. 3 and 7. The linear regression lines were drawn through all points of the graphs

To determine whether there is an association between increased TRPC expression and coronary artery pathology, we performed the Pearson Product Moment Correlation analysis (SigmaPlot 13). TRPC6 expression and histamine-induced active tension (Fig. 4) as well as TRPC1 expression and atherosclerosis associations (Fig. 3) were analyzed. We found that the Pearson Product Moment correlation coefficients amounted to 0.78 and 0.86 for the TRPC6-active tension data sets and the TRPC1-percent wall coverage data sets, respectively, indicating a strong correlation between these pairs of variables (Fig. 8).

### TRPC1 immunostaining is predominantly found in macrophage-rich atheroma areas

**Fig. 9:**
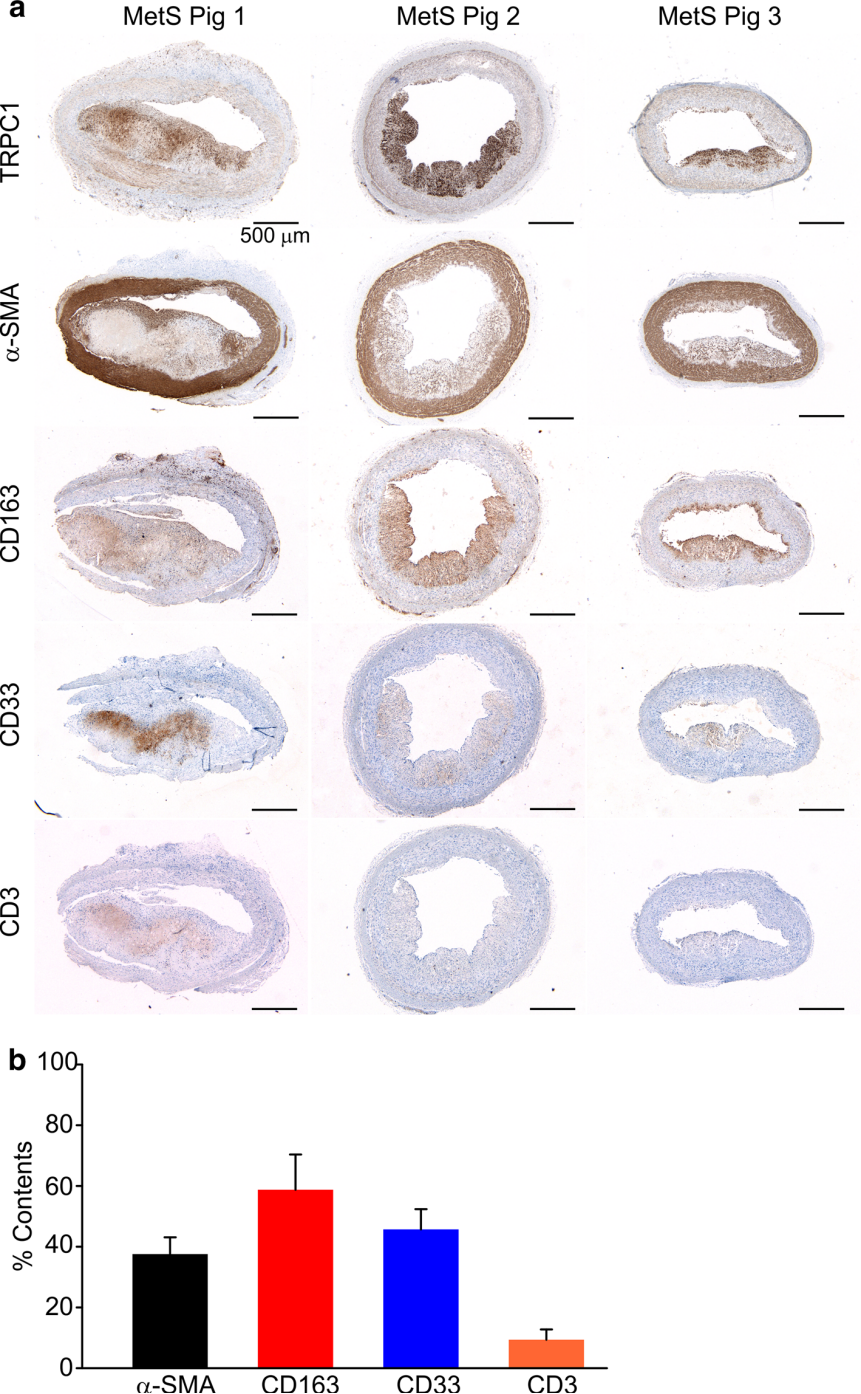
Immunostaining of MetS coronary artery sections from three representative MetS pigs. **a** The sections were stained with the TRPC1, α-smooth muscle actin (SMA), CD163, CD33, or CD3 antibodies. *Scale bars* 500 μm. The “MetS-TRPC1 antibody” image in Suppl. Figure 2 is identical to the image of “MetS Pig 2-TRPC1” in **a**. **b** Percent contents of α-smooth muscle actin (SMA), CD163, CD33, or CD3 in atherosclerotic lesions

The fact that the strongest TRPC1 immunostaining was observed in atheromas rather than in the medial layer prompted us to hypothesize that TRPC1 expression may be localized to the proliferating SMCs in atheromas. To test this hypothesis, SMCs in the adjacent MetS sections were stained with an α-smooth muscle actin (SMA) antibody. Unexpectedly, we found that although TRPC1 immunostaining was present in some neointimal SMCs of atheromas, it was stronger in the plaque areas having less SMA immunoreactivity (Fig. 9; Suppl. Figure 3). Therefore, we next stained the adjacent sections of coronary arteries from MetS pigs for markers of macrophages (CD163 and CD33) and T cells (CD3) and compared staining patterns with that of TRPC1. It was noticeable that TRPC1 staining was predominantly observed in the segments of atheromas with robust CD163 and CD33 immunostaining, and scant CD3 immunostaining (Fig. 9a; Suppl. Figure 3), indicating atheroma’s TRPC1 immunostaining is not only found in neointimal SMCs but also strongly associated with macrophages. Figure 9b shows the phenotyping of atherosclerotic lesions. The mean % content of α-SMA, CD163, CD33, and CD3 immunoreactivities amounted to 37.5 ± 5.6, 58.8 ± 11.6, 45.7 ± 6.7, and 9.4 ± 3.4%, respectively (*n* = 6, in each case).

### In vivo dominant-negative TRPC6 cDNA construct delivery

**Fig. 10:**
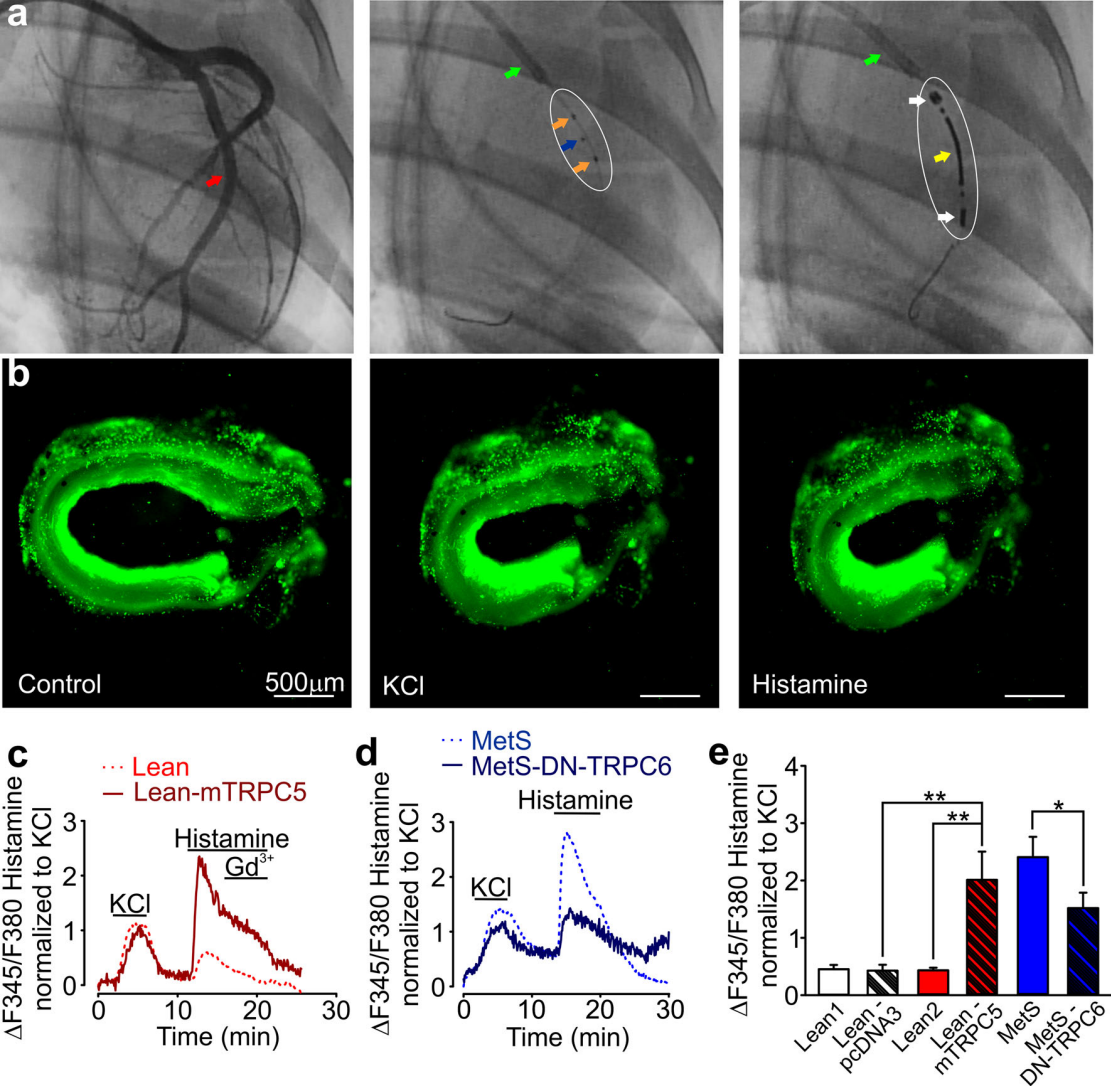
In vivo electroporation in Lean and MetS pigs. **a** X-ray angiographic images demonstrating the steps of in vivo electroporation procedure in pig coronary arteries. *Left image* a MetS coronary artery tree (*red arrow*) was visualized using the contrast reagent injected via the guiding catheter. *Middle image* the placement of the injection catheter into the circumflex artery of the same pig. The *green arrow* shows the guiding catheter. The *blue arrow* shows the injection microneedle. The *orange arrows* show the two radio-opaque markers. The *white circle* shows the approximate position of the injection catheter. *Right image* the placement of the electroporation catheter in the same pig artery. The *green arrow* shows the guiding catheter. The *white arrow* shows the proximal and distal electrodes. The *yellow arrows* show the central electrode. The *white circle* shows an approximate position of the electroporation catheter. **b** Fluorescence images of a Lean coronary artery ring from an in vivo injected and then electroporated pig, which were obtained at an excitation wavelength of 380 nm (*left panel* control; *middle panel* under 70 mM KCl; *right panel* under 40 μM histamine; Lean-mTRPC5). **c**, **d** Sample traces of 70 mM KCl and 40 μM histamine-induced [Ca^2+^]_i_ changes in coronary artery rings (**c** mTRPC5; **d** DN-TRPC6). **e** Summary data are shown for the tested rings (Lean 1, *n* = 11; Lean-pcDNA3, *n* = 4; Lean 2, *n* = 6; Lean-mTRPC5, *n* = 6; MetS, *n* = 7; MetS-DN-TRPC6, *n* = 6). The peak amplitudes of [Ca^2+^]_i_ changes in the presence of histamine (40 µM) are normalized to KCl (70 mM). *pcDNA3* the empty vector, *mTRPC5* mouse TRPC5, *DN-TRPC6* dominant-negative TRPC6. *P < 0.05; **P < 0.01. The unpaired *t* test was used to determine whether there is a statistically significant difference between the indicated two data sets

The correlation between TRPC6 expression and histamine-induced coronary contractions prompted us to investigate whether decreasing coronary TRPC6 activity would reduce histamine-induced [Ca^2+^]_i_ transients in MetS pig coronary arteries. In vivo gene transfer of the dominant-negative (DN) TRPC6 construct was performed into coronary artery walls in beating hearts. Figure 10a shows sample angiographic images obtained during the intracoronary placement of the injection and electroporation catheters inside of a circumflex coronary artery in vivo (see “Materials and methods” for details). We found that electroporated coronary arteries were fragile (Fig. 10b), complicating isometric tension experiments with the electroporated rings. Therefore, we focused on fluorescence imaging to compare the peak amplitudes of histamine-induced [Ca^2+^]_i_ transients in the in vivo injected/electroporated and control rings adjacent to the electroporated area, which were isolated from the same pig. [Ca^2+^]_i_ transients are critical for triggering coronary smooth muscle cell contractions. In control experiments, the Lean pig coronary artery rings that were in vivo injected with pcDNA3 empty vector and then intracoronary electroporated exhibited normalized histamine-induced [Ca^2+^]_i_ transients that were not significantly different from those observed in non-electroporated rings (Fig. 10c, e). While the empty vector had no effect, we confirmed that there was no non-specific effect of the electroporation and instrumentation by over expressing another histamine-regulated channel, TRPC5. The Lean pig coronary artery rings in vivo injected/electroporated with mTRPC5-pcDNA3 exhibited significantly greater normalized histamine-induced [Ca^2+^]_i_ transients compared to those observed in pcDNA3-injected/electroporated rings (Fig. 10c, e). Gd^3+^ (100 μM) caused only small or no inhibition of histamine-induced [Ca^2+^]_i_ transients in the tested rings (Fig. 10c), likely due to poor permeability of pig coronary artery wall to Gd^3+^. Importantly, we found that normalized histamine-induced [Ca^2+^]_i_ transients were significantly decreased in MetS pig coronary artery rings in vivo injected/electroporated with a dominant-negative (DN) TRPC6 construct as compared to those observed in control rings (Fig. 10d, e).

### Aldosterone treatment increases histamine-induced contractions of organ-cultured coronary arteries in a mineralocorticoid receptor-dependent manner

**Fig. 11:**
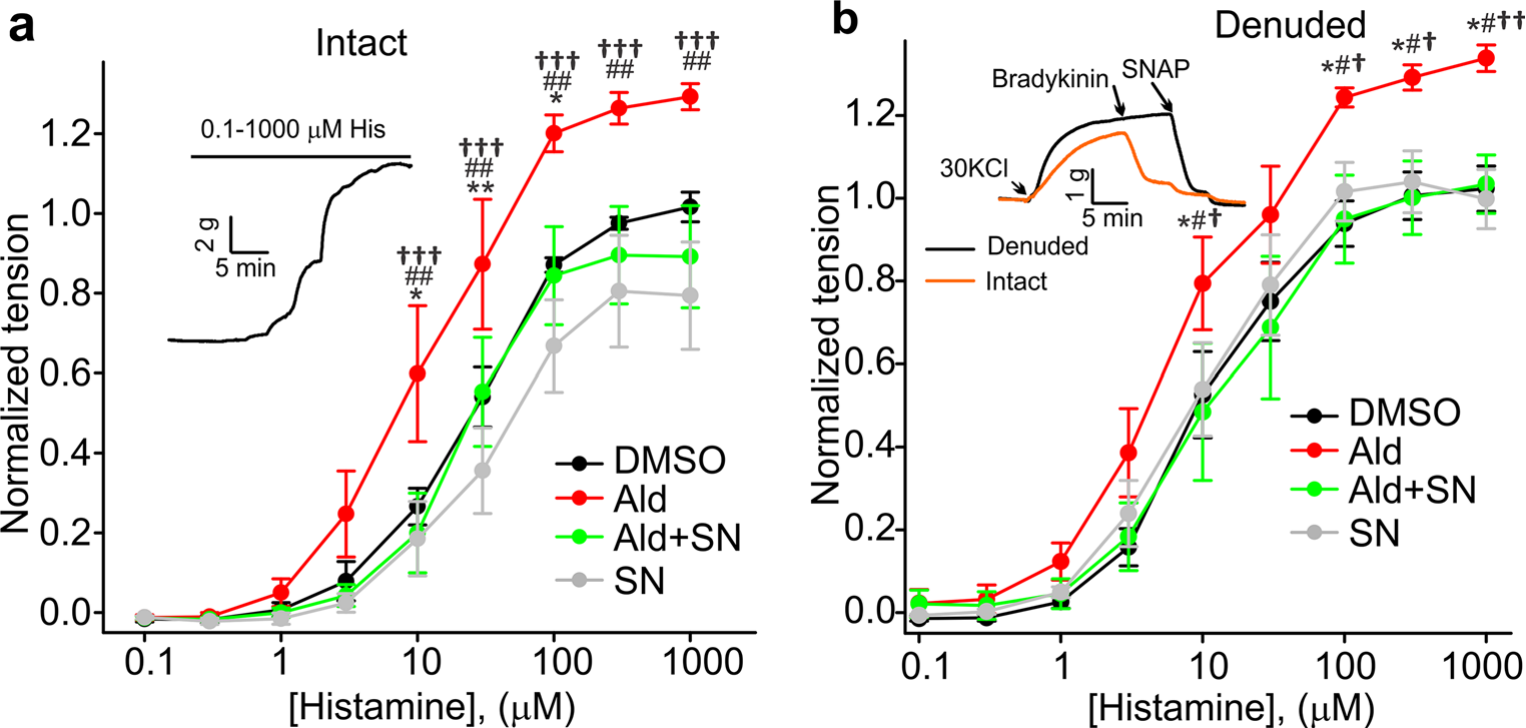
Effect of aldosterone and spironolactone treatment on organ-cultured coronary arteries. **e**, **f** Concentration–response curves for histamine in intact (**a**, *n* = 7) and denuded (**b**, *n* = 6) organ-cultured rings treated with DMSO, aldosterone, aldosterone plus spironolactone, or spironolactone for seven days (*Ald vs. DMSO; ^#^Ald vs. Ald + SN; ^†^Ald vs. SN). Sample traces are shown in *insets*. *P < 0.05, **P < 0.01, ^†^ *P* < 0.05, ^††^ *P* < 0.01, ^†††^ *P* < 0.001, ^#^ *P* < 0.05, ^##^ *P* < 0.01. The two-way ANOVA test followed by the Student–Newman–Keuls post hoc test

We previously reported that TRPC6 expression was upregulated in coronary arteries of MetS pigs [16]; and that plasma aldosterone was elevated in MetS Ossabaw pigs [2]. It was also reported that that aldosterone treatment leads to increased TRPC6 expression in arterial smooth muscle cells [3]. To provide further evidence supporting the hypothesis that TRPC6 upregulation correlates with increased pig coronary artery contractility, we next employed the in vitro organ-culture model. Using quantitative RT-PCR, we confirmed that aldosterone treatment increased TRPC6 expression in organ-cultured Lean pig coronary artery rings (Suppl. Figure 4). Then, we assessed whether aldosterone treatment would increase coronary contractility, and whether inhibition of the mineralocorticoid receptor by spironolactone would prevent this effect. Four groups of Lean coronary artery rings were initiated as follows: (1) DMSO (vehicle control) group; (2) aldosterone (100 nM) group; (3) aldosterone (100 nM) plus spironolactone (10 μM) group; and (4) spironolactone (10 μM) group. The rings (3 mm long) were organ-cultured for 7 days without fetal bovine serum to avoid any possible contribution of growth factors and other vasoactive molecules present in the fetal bovine serum; and the histamine-induced contractility of the rings was measured using a wire myograph. Figure 11 shows that the contractions induced by 10, 100, 300, and 1000 µM histamine were significantly stronger in both intact (Fig. 11a, *n* = 7) and denuded (Fig. 11b, *n* = 6) rings treated with aldosterone as compared to those treated with DMSO and spironolactone. Aldosterone-induced increases in coronary contractility were comparable in both denuded and intact rings, arguing against any role of the endothelium in mediating the aldosterone-induced contractility changes. Importantly, the “aldosterone plus spironolactone”-treated rings exhibited contraction amplitudes similar to those observed in the DMSO-treated and spironolactone-treated groups, but significantly smaller than those found in the aldosterone-treated group. These data indicate that inhibition of the mineralocorticoid receptor by spironolactone prevents aldosterone-dependent upregulation of histamine-induced coronary contractility.

### Monocytes adhesion to the endothelium is regulated by aldosterone in a mineralocorticoid receptor-dependent manner

**Fig. 12:**
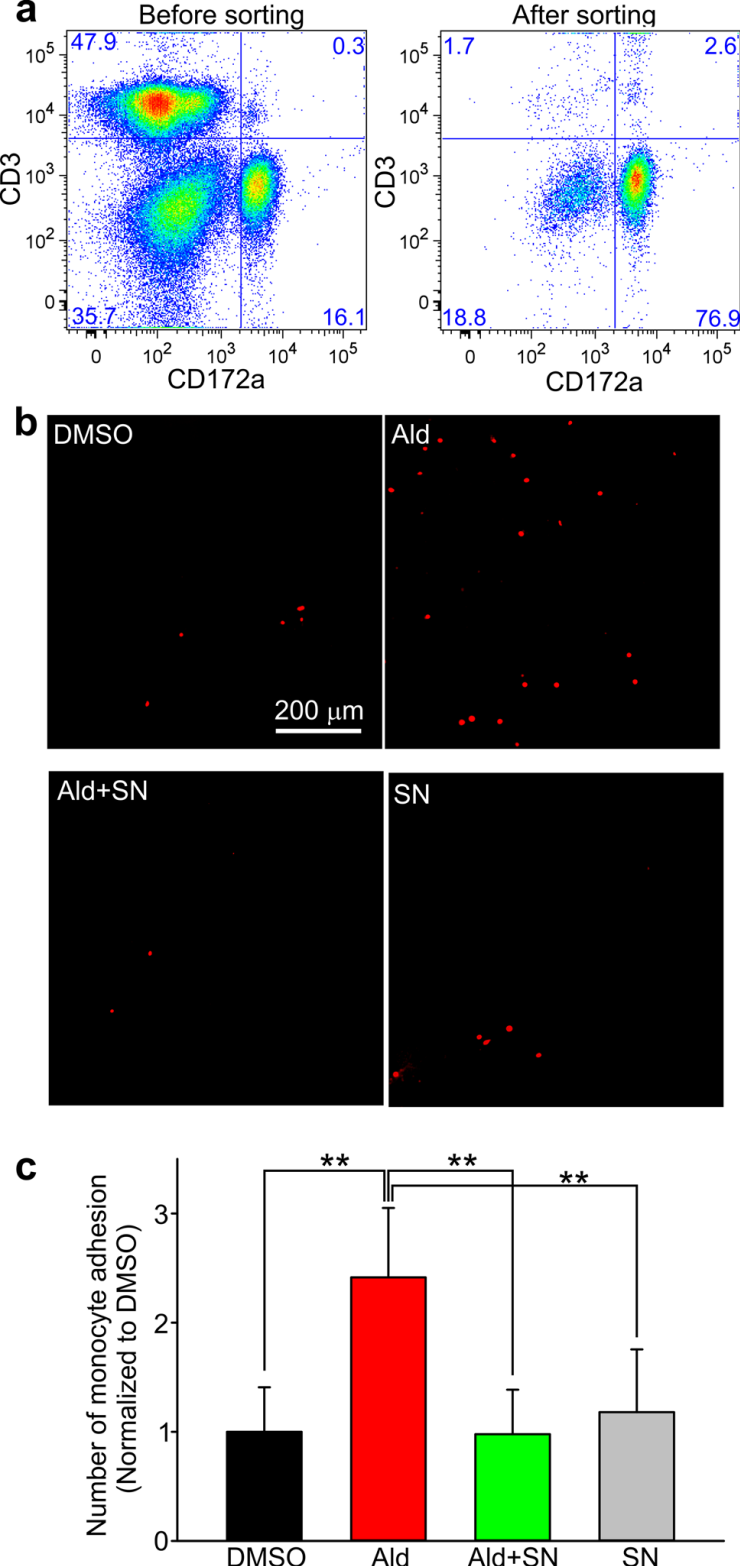
Monocytes adhesion assay. **a** Representative images show flow cytometry sorts of peripheral blood mononuclear cells before and after monocyte enrichment. The cells were stained with pig CD3 and CD172a antibodies. Images show a marked increase in monocyte populations and T-cell depletion. **b** Images of monocytes adhesion to the endothelium after an incubation for 2 h with coronary artery strips pretreated with DMSO, aldosterone (Ald), aldosterone plus spironolactone (Ald + SN) and spironolactone (SN) for 48 h. The monocytes were loaded with the dye of SNARF-1 and seen as *red spots*. *Bar* 200 μm. **c** Summary for data shown in **b**. **P < 0.01. The one-way repeated measures ANOVA test followed by the Holm–Sidak post hoc test

The adhesion of monocytes to the endothelium of coronary arteries and their subsequent extravasation constitute the earliest steps of atherosclerosis progression. To investigate the role of aldosterone in regulating monocyte adhesion to the endothelium of Lean pig coronary arteries, we again used the organ-cultured coronary arteries. Again, four test groups were initiated and treated for 48 h with (1) DMSO; (2) aldosterone; (3) aldosterone plus spironolactone; and (4) spironolactone. Coronary artery strips were used in these experiments. Peripheral blood monocytes were isolated using flow cytometry. Figure 12a shows that the degree of monocyte enrichment exceeded 75%, about five times higher than the initial monocyte count (16.1%). The pig monocytes exhibited low levels of adhesion to the endothelium of DMSO and spironolactone-treated coronary arteries. Only a few adherent cells were observed in these experiments (Fig. 12b). Conversely, aldosterone treatment increased monocytes adhesion to coronary artery strips by 2.4 ± 0.6 fold. Spironolactone treatment potently inhibited aldosterone-mediated monocyte adhesion to the coronary endothelium (Fig. 12c).

### TRPC1 is expressed in macrophages differentiated from pig peripheral blood monocytes

We next investigated whether the expression level of TRPC1 is distinct in differentiated macrophages obtained from the Lean, MetS, and MetS-SN groups. Pig peripheral blood mononuclear cells were isolated and differentiated into macrophages by culturing in the presence of the L929 conditioned medium. TRPC1 mRNA expression was quantified in the differentiated macrophages. TRPC1 was equally expressed in the macrophages across the tested groups (Suppl. Figure 5).

## Discussion

In this study, we investigated the roles of TRPC channels in MetS coronary arteries and determined whether spironolactone exhibits any anti-atherosclerotic potential in the Ossabaw pig model of diet-induced metabolic syndrome. Our principal new findings include: (1) TRPC1 is highly expressed in the macrophages residing in the atheroma of metabolic syndrome coronary arteries; (2) elevated TRPC6 is mostly found in the contractile coronary SMCs of the medial layer but not in proliferative neointimal SMCs or macrophages of atheroma; (3) histamine-induced [Ca^2+^]_i_ transients are significantly decreased in the MetS coronary artery segments after in vivo targeted DN-TRPC6 delivery; and (4) long-term spironolactone treatment significantly decreases the protein expression level of TRPC1 and TRPC6 channels in coronary arteries associated with reduced native atherosclerosis and vasoconstriction.

Upregulation of TRPC1 expression in neointimal SMCs is critical for occlusive coronary artery disease progression in the pig model of neointimal hyperplasia after vascular injury via balloon angioplasty [33]. Surprisingly, we found that in contrast to the vascular injury model, the diet-induced pig model of MetS-associated atherosclerosis showed that the TRPC1 protein is predominantly expressed in macrophages residing in the cores of atheromas (Suppl. Figure 3), although TRPC1 immunostaining is also observed in some neointimal SMCs. Thus, our data underscore the importance of macrophage TRPC1 expression in coronary atherosclerosis in MetS.

Unlike TRPC1, TRPC6 expression was greater in the medial layer of MetS coronary arteries. Both histamine-induced contractions and [Ca^2+^]_i_ transients are increased in coronary arteries from the MetS group that exhibited an increased TRPC6 protein level in the medial layer. These data support our overall hypothesis that higher TRPC6 expression in the smooth muscle layer correlates with increased coronary contractility.

Coronary arteries from spironolactone-treated MetS pigs exhibited milder atherosclerosis and reduced collagen content and spasticity compared to age-matched control MetS coronary arteries did. Thus, our findings provide evidence that spironolactone attenuates MetS-associated coronary atherosclerosis. These data are consistent with two previous reports indicating that another mineralocorticoid receptor inhibitor, eplerenone, and aldosterone synthase inhibitors are effective at slowing plaque growth in a mouse ApoE^−/−^ model of aortic atherosclerosis [20, 31] and a recent initial clinical study reporting that spironolactone may decrease atherosclerosis in humans [37]. Importantly, in this study, we utilized the diet-induced pig model of MetS-associated coronary atherosclerosis, which better represents the human setting than the mouse ApoE knock-out/cholesterol handling-dependent model of atherosclerosis [34].

Previously, we reported that TRPC1 and TRPC6 expression is markedly elevated in metabolic syndrome pig coronary arteries [16]. The promoter regions of TRPC1 and TRPC6 possess half-site glucocorticoid/mineralocorticoid response elements [26] driving the expression of these TRPC channels in metabolic syndrome, characterized by elevated plasma aldosterone [2]. Our new data indicate that one of the mechanisms underlying spironolactone’s beneficial actions in atherosclerosis may include decreased TRPC channel expression in the coronary artery wall. Specifically, spironolactone-dependent reduction of TRPC1 in atheroma was strongly associated with a marked decrease of atherosclerosis, whereas spironolactone-dependent downregulation of medial TRPC6 was associated with decreased histamine-induced contractility and reduced [Ca^2+^]_i_ transients in the medial layer. Consistently, we found that in vivo targeted delivery of the DN-TRPC6 construct into the MetS coronary artery wall using an in vivo intracoronary electroporation approach resulted in decreased histamine-induced [Ca^2+^]_i_ transients in the MetS coronary artery segments. Collectively, this supports the hypothesis that TRPC6 upregulation leads to increased coronary contractility. However, the limitation of this study is that coronary artery reactivity is assessed only using the in vitro approaches in coronary artery rings. Although these coronary artery ring data were important for establishing the critical correlation between TRPC6 expression and coronary artery contractility in diet-induced metabolic syndrome, future experiments will be needed to confirm the present in vitro reactivity results using the in vivo coronary histaminergic provocation test.

Macrophage accumulation in the coronary intima plays a critical role during atherosclerotic plaque formation and growth [14, 30]. However, the mechanism of how macrophage TRPC1 expression promotes coronary atherosclerosis progression remains unclear. We found that TRPC1 and macrophage immunostaining were pronounced in MetS atheromas and reduced in MetS-SN atheromas. Since elevated aldosterone is a feature of metabolic syndrome, we hypothesized that higher TRPC1 immunostaining in MetS atheromas may be due to an aldosterone-dependent increase in monocyte adhesion to the coronary endothelium and that spironolactone may decrease monocyte adhesion. Indeed, it was reported that endothelial intercellular adhesion molecule (ICAM1) gene and protein expression was upregulated in human coronary artery endothelial cells by aldosterone in a mineralocorticoid receptor-dependent manner [7], leading to increased adhesion of leukocytes. To test our hypothesis, we used the organ-cultured coronary artery rings from Lean pigs. We treated the organ-cultured rings with aldosterone and found that aldosterone did increase adhesion of monocytes to pig coronary artery endothelium (Fig. 12). Importantly, spironolactone antagonized this effect. Therefore, we speculate that spironolactone may slow down atherosclerosis progression by reducing TRPC1-positive monocyte adhesion to the coronary endothelium and their subsequent extravasation.

Spironolactone inhibits microsomal cholesterol 7α-hydroxylase activity and increases bile salt-independent bile flow (choleresis) in the liver, resulting in increased cholesterol excretion into the bile [42]. However, subsequent clinical studies revealed controversial findings, indicating that spironolactone may or may not affect total cholesterol level in human subjects [9, 17, 39, 48]. Elevated levels of plasma cholesterol are strongly associated with atherosclerosis development [4]. Therefore, we investigated spironolactone’s effect on total plasma cholesterol level in the Ossabaw pig model. Our data indicate that spironolactone does not significantly reduce plasma cholesterol concentration in the Ossabaw pig model of MetS. Therefore, we propose that the observed effects of spironolactone in our study are unlikely to be due to a reduction of total plasma cholesterol concentration in the MetS-SN pigs.

Long-term spironolactone treatment in MetS pigs prevents MetS-associated endothelial dysfunction. These data are consistent with a recent report that eplerenone, another antagonist of the mineralocorticoid receptor, prevented endothelial dysfunction associated with diet-induced obesity in C57BL/6 mice [44]. Schafer et al. [44] provided evidence that eplerenone-mediated inhibition of endothelial mineralocorticoid receptors results in a reduction of pro-oxidative p22phox and p40phox gene expression and an increase in expression of antioxidative genes, such as glutathione peroxidase-1 and superoxide dismutase, counteracting aldosterone-dependent reactive oxygen species generation in endothelial cells. We speculate that a similar mechanism may underlie spironolactone’s effects in our pig model of metabolic syndrome.

The major physiological function of aldosterone in the human body is to regulate Na^+^ and K^+^ plasma concentrations. Aldosterone acting via the mineralocorticoid receptor in the principal epithelial cells of the kidney collecting duct causes Na^+^ retention, which is important for regulating blood volume. However, excessive aldosterone-dependent Na^+^ retention results in hypertension. Spironolactone, a competitive inhibitor of the mineralocorticoid receptors, is widely prescribed to treat hypertension due to the abnormal Na^+^ retention by the kidney. In this study, we did find that the mean systolic blood pressure was elevated in the MetS pigs. However, the mean systolic blood pressure in MetS-SN pigs was not significantly different from its mean value in MetS pigs. Given the fact that neither blood pressure nor plasma Na^+^ concentration was significantly different in MetS and MetS-SN groups (Table 1), these data argue against the notion that spironolactone action on the kidney underlies the anti-atherosclerotic effect of this drug. We propose that mineralocorticoid receptor antagonism by spironolactone in the coronary artery wall rather than in the kidney contributes to the improvements observed in the MetS-SN group.

The combined reduction in arterial pressure and plasma cholesterol, although both not statistically significant, may synergize to attenuate atheroma. We contend, however, that this combination of systemic factors is unlikely to be the underlying cause for the reduced lesion size. We propose that the direct vascular wall signaling plays the major role in atheroma reduction.

## Conclusions

This study demonstrates that long-term mineralocorticoid receptor blockade with spironolactone decreased coronary TRPC expression, atherosclerosis, and hypercontractility in the Ossabaw pig model of metabolic syndrome without impacting obesity or glucose tolerance. Targeted down-regulation of TRPC6 by intracoronary application of a dominant-negative construct provides additional evidence for the key role of TRPC6 in hypercontractility of MetS coronary arteries. TRPC1-positive macrophages present in the coronary artery wall of MetS pigs are associated with advanced atherosclerotic lesions.

## Electronic supplementary material

Below is the link to the electronic supplementary material.

**Suppl. Figure** **1** Images of the coronary artery sections shown in Fig. 1c-g, but taken with a 4-x objective. The black arrow indicate the small atheroma in a coronary artery ring from a Lean pig (TIFF 10645 kb)

**Suppl. Figure** **2** Images of the coronary artery sections shown in Fig. 4a, but taken with 4-x objective. The MetS-TRPC1 antibody image is identical to the image “MetS Pig 2-TRPC1 antibody” in Fig. 9 (TIFF 17152 kb)

**Suppl. Figure** **3** Adjacent coronary artery sections from the same MetS pig stained with the TRPC1, α-SMA, CD3, or CD163 antibodies (TIFF 30617 kb)

**Suppl. Figure** **4** Quantitative RT-PCR analysis of TRPC6 expression in organ-cultured Lean pig coronary artery rings. The rings were cultured for 36 h in the presence of either the vehicle, aldosterone (100 nM), aldosterone (100 nM) plus spironolactone (1 μM), or spironolactone (1 μM). The TRPC6 expression was normalized to the 18S rRNA expression level and then to the vehicle control. P = 0.007. The Kruskal–Wallis One-Way Analysis of Variance on Ranks test followed by the post hoc all pairwise multiple comparison Dunn’s test (TIFF 620 kb)

**Suppl. Figure** **5** Quantitative RT-PCR analysis of TRPC1 expression in the cultured macrophages differentiated from peripheral blood mononuclear cells. Total mRNA was isolated from the cultured macrophage and then reverse-transcribed into cDNA. The primer pairs were purchased from Qiagen. The TRPC1 expression was normalized to the β_2_ microglobulin expression level. No significant difference was observed among the treatment groups (P = 0.073, the Kruskal–Wallis One-Way Analysis of Variance on Ranks test) (TIFF 641 kb)

## References

[1] Albert AP, Saleh SN, Large WA (2009). Identification of canonical transient receptor potential (TRPC) channel proteins in native vascular smooth muscle cells. Curr Med Chem.

[2] Alloosh M, Pratt JH, Sturek M, Basile D (2008). Elevated renin and enhanced adrenal steroidogenesis in the Ossabaw swine model of the metabolic syndrome. FASEB J.

[3] Bae YM, Kim A, Lee YJ, Lim W, Noh YH, Kim EJ, Kim J, Kim TK, Park SW, Kim B, Cho SI, Kim DK, Ho WK (2007). Enhancement of receptor-operated cation current and TRPC6 expression in arterial smooth muscle cells of deoxycorticosterone acetate-salt hypertensive rats. J Hypertens.

[4] Bender SB, de Beer VJ, Tharp DL, Bowles DK, Laughlin MH, Merkus D, Duncker DJ (2016). Severe familial hypercholesterolemia impairs the regulation of coronary blood flow and oxygen supply during exercise. Basic Res Cardiol.

[5] Bernini G, Galetta F, Franzoni F, Bardini M, Taurino C, Bernardini M, Ghiadoni L, Bernini M, Santoro G, Salvetti A (2008). Arterial stiffness, intima-media thickness and carotid artery fibrosis in patients with primary aldosteronism. J Hypertens.

[6] Bochud M, Nussberger J, Bovet P, Maillard MR, Elston RC, Paccaud F, Shamlaye C, Burnier M (2006). Plasma aldosterone is independently associated with the metabolic syndrome. Hypertension.

[7] Caprio M, Newfell BG, la Sala A, Baur W, Fabbri A, Rosano G, Mendelsohn ME, Jaffe IZ (2008). Functional mineralocorticoid receptors in human vascular endothelial cells regulate intercellular adhesion molecule-1 expression and promote leukocyte adhesion. Circ Res.

[8] Chakraborty S, Berwick ZC, Bartlett PJ, Kumar S, Thomas AP, Sturek M, Tune JD, Obukhov AG (2011). Bromoenol lactone inhibits voltage-gated Ca^2+^ and transient receptor potential canonical channels. J Pharmacol Exp Ther.

[9] Chapman N, Dobson J, Wilson S, Dahlof B, Sever PS, Wedel H, Poulter NR (2007). Effect of spironolactone on blood pressure in subjects with resistant hypertension. Hypertension.

[10] Chen X, Egly C, Riley AM, Li W, Tewson P, Hughes TE, Quinn AM, Obukhov AG (2014). PKC-dependent phosphorylation of the H1 histamine receptor modulates TRPC6 activity. Cells.

[11] Chen X, Li W, Hiett SC, Obukhov AG (2016). Novel roles for K_v_7 channels in shaping histamine-induced contractions and bradykinin-dependent relaxations in pig coronary arteries. PLoS One.

[12] Chen X, Li W, Riley AM, Soliman M, Chakraborty S, Stamatkin CW, Obukhov AG (2017). Molecular determinants of the sensitivity to G_q/11_-phospholipase C-dependent gating, Gd^3+^ potentiation, and Ca^2+^ permeability in the transient receptor potential canonical type 5 (TRPC5) channel. J Biol Chem.

[13] Chiang JK, Chen CL, Tseng FY, Chi YC, Huang KC, Yang WS (2015). Higher blood aldosterone level in metabolic syndrome is independently related to adiposity and fasting plasma glucose. Cardiovasc Diabetol.

[14] Cochain C, Zernecke A (2015). Macrophages and immune cells in atherosclerosis: recent advances and novel concepts. Basic Res Cardiol.

[15] de Rita O, Hackam DG, Spence JD (2012). Effects of aldosterone on human atherosclerosis: plasma aldosterone and progression of carotid plaque. Can J Cardiol.

[16] Edwards JM, Neeb ZP, Alloosh MA, Long X, Bratz IN, Peller CR, Byrd JP, Kumar S, Obukhov AG, Sturek M (2010). Exercise training decreases store-operated Ca^2+^ entry associated with metabolic syndrome and coronary atherosclerosis. Cardiovasc Res.

[17] Falch DK, Schreiner A (1983). The effect of spironolactone on lipid, glucose and uric acid levels in blood during long-term administration to hypertensives. Acta Med Scand.

[18] Forman MB, Oates JA, Robertson D, Robertson RM, Roberts LJ, Virmani R (1985). Increased adventitial mast cells in a patient with coronary spasm. N Engl J Med.

[19] Frostegard J (2013). Immunity, atherosclerosis and cardiovascular disease. BMC Med.

[20] Gamliel-Lazarovich A, Gantman A, Coleman R, Jeng AY, Kaplan M, Keidar S (2010). FAD286, an aldosterone synthase inhibitor, reduced atherosclerosis and inflammation in apolipoprotein E-deficient mice. J Hypertens.

[21] Gasper WJ, Jimenez CA, Walker J, Conte MS, Seward K, Owens CD (2013). Adventitial nab-rapamycin injection reduces porcine femoral artery luminal stenosis induced by balloon angioplasty via inhibition of medial proliferation and adventitial inflammation. Circ Cardiovasc Interv.

[22] Ginsburg R, Bristow MR, Davis K, Dibiase A, Billingham ME (1984). Quantitative pharmacologic responses of normal and atherosclerotic isolated human epicardial coronary arteries. Circulation.

[23] Grundy SM, Brewer HB, Cleeman JI, Smith SC, Lenfant C (2004). Definition of metabolic syndrome: report of the National Heart, Lung, and Blood Institute/American Heart Association conference on scientific issues related to definition. Circulation.

[24] Hiett SC, Owen MK, Li W, Chen X, Riley A, Noblet J, Flores S, Sturek M, Tune JD, Obukhov AG (2014). Mechanisms underlying capsaicin effects in canine coronary artery: implications for coronary spasm. Cardiovasc Res.

[25] Hillaert MA, Lentjes EG, Kemperman H, van der Graaf Y, Nathoe HM, Beygui F, Montalescot G, Doevendans PA, Wassink AM, van Belle E (2013). Aldosterone, atherosclerosis and vascular events in patients with stable coronary artery disease. Int J Cardiol.

[26] Hu G, Oboukhova EA, Kumar S, Sturek M, Obukhov AG (2009). Canonical transient receptor potential channels expression is elevated in a porcine model of metabolic syndrome. Mol Endocrinol.

[27] Hung MJ, Hu P, Hung MY (2014). Coronary artery spasm: review and update. Int J Med Sci.

[28] Ivanes F, Susen S, Mouquet F, Pigny P, Cuilleret F, Sautiere K, Collet JP, Beygui F, Hennache B, Ennezat PV, Juthier F, Richard F, Dallongeville J, Hillaert MA, Doevendans PA, Jude B, Bertrand M, Montalescot G, van Belle E (2012). Aldosterone, mortality, and acute ischaemic events in coronary artery disease patients outside the setting of acute myocardial infarction or heart failure. Eur Heart J.

[29] Jaisser F, Farman N (2016). Emerging roles of the mineralocorticoid receptor in pathology: toward new paradigms in clinical pharmacology. Pharmacol Rev.

[30] Jansen MF, Hollander MR, van Royen N, Horrevoets AJ, Lutgens E (2016). CD40 in coronary artery disease: a matter of macrophages?. Basic Res Cardiol.

[31] Keidar S, Hayek T, Kaplan M, Pavlotzky E, Hamoud S, Coleman R, Aviram M (2003). Effect of eplerenone, a selective aldosterone blocker, on blood pressure, serum and macrophage oxidative stress, and atherosclerosis in apolipoprotein E-deficient mice. J Cardiovasc Pharmacol.

[32] Kidambi S, Kotchen JM, Grim CE, Raff H, Mao J, Singh RJ, Kotchen TA (2007). Association of adrenal steroids with hypertension and the metabolic syndrome in blacks. Hypertension.

[33] Kumar B, Dreja K, Shah SS, Cheong A, Xu SZ, Sukumar P, Naylor J, Forte A, Cipollaro M, McHugh D, Kingston PA, Heagerty AM, Munsch CM, Bergdahl A, Hultgardh-Nilsson A, Gomez MF, Porter KE, Hellstrand P, Beech DJ (2006). Upregulated TRPC1 channel in vascular injury in vivo and its role in human neointimal hyperplasia. Circ Res.

[34] Libby P (2015). Murine “model” monotheism: an iconoclast at the altar of mouse. Circ Res.

[35] McGraw AP, Bagley J, Chen WS, Galayda C, Nickerson H, Armani A, Caprio M, Carmeliet P, Jaffe IZ (2013). Aldosterone increases early atherosclerosis and promotes plaque inflammation through a placental growth factor-dependent mechanism. J Am Heart Assoc.

[36] Modena MG, Aveta P, Menozzi A, Rossi R (2001). Aldosterone inhibition limits collagen synthesis and progressive left ventricular enlargement after anterior myocardial infarction. Am Heart J.

[37] Moss ME, Jaffe IZ (2015). Mineralocorticoid receptors in the pathophysiology of vascular inflammation and atherosclerosis. Front Endocrinol (Lausanne).

[38] Mottillo S, Filion KB, Genest J, Joseph L, Pilote L, Poirier P, Rinfret S, Schiffrin EL, Eisenberg MJ (2010). The metabolic syndrome and cardiovascular risk a systematic review and meta-analysis. J Am Coll Cardiol.

[39] Nakhjavani M, Hamidi S, Esteghamati A, Abbasi M, Nosratian-Jahromi S, Pasalar P (2009). Short term effects of spironolactone on blood lipid profile: a 3-month study on a cohort of young women with hirsutism. Br J Clin Pharmacol.

[40] Nilius B (2007). TRP channels in disease. Biochim Biophys Acta.

[41] Obukhov AG, Nowycky MC (2008). TRPC5 channels undergo changes in gating properties during the activation-deactivation cycle. J Cell Physiol.

[42] Ruiz ML, Villanueva SS, Luquita MG, Sanchez-Pozzi EJ, Crocenzi FA, Pellegrino JM, Ochoa JE, Vore M, Mottino AD, Catania VA (2005). Mechanisms involved in spironolactone-induced choleresis in the rat. Role of multidrug resistance-associated protein 2. Biochem Pharmacol.

[43] Sakata Y, Komamura K, Hirayama A, Nanto S, Kitakaze M, Hori M, Kodama K (1996). Elevation of the plasma histamine concentration in the coronary circulation in patients with variant angina. Am J Cardiol.

[44] Schafer N, Lohmann C, Winnik S, van Tits LJ, Miranda MX, Vergopoulos A, Ruschitzka F, Nussberger J, Berger S, Luscher TF, Verrey F, Matter CM (2013). Endothelial mineralocorticoid receptor activation mediates endothelial dysfunction in diet-induced obesity. Eur Heart J.

[45] Seidler RW, Allgauer S, Ailinger S, Sterner A, Dev N, Rabussay D, Doods H, Lenter MC (2005). In vivo human MCP-1 transfection in porcine arteries by intravascular electroporation. Pharm Res.

[46] Shimokawa H, Tomoike H, Nabeyama S, Yamamoto H, Araki H, Nakamura M, Ishii Y, Tanaka K (1983). Coronary artery spasm induced in atherosclerotic miniature swine. Science.

[47] Spinas E, Kritas SK, Saggini A, Mobili A, Caraffa A, Antinolfi P, Pantalone A, Tei M, Speziali A, Saggini R, Conti P (2014). Role of mast cells in atherosclerosis: a classical inflammatory disease. Int J Immunopathol Pharmacol.

[48] Studen KB, Sebestjen M, Pfeifer M, Prezelj J (2011). Influence of spironolactone treatment on endothelial function in non-obese women with polycystic ovary syndrome. Eur J Endocrinol.

[49] Yamagishi M, Miyatake K, Tamai J, Nakatani S, Koyama J, Nissen SE (1994). Intravascular ultrasound detection of atherosclerosis at the site of focal vasospasm in angiographically normal or minimally narrowed coronary segments. J Am Coll Cardiol.

[50] Yun BH, Chon SJ, Lee YJ, Han EJ, Cho S, Choi YS, Lee BS, Seo SK (2015). Association of metabolic syndrome with coronary atherosclerosis in non-diabetic postmenopausal women. Climacteric.

