# Supplementary figures and images for: Long-term spironolactone treatment reduces coronary TRPC expression, vasoconstriction, and atherosclerosis in metabolic syndrome pigs
Source: Basic Res Cardiol. 2017 Jul 29;112(5):54. doi: 10.1007/s00395-017-0643-0 (PMC5534204; doi:10.1007/s00395-017-0643-0)

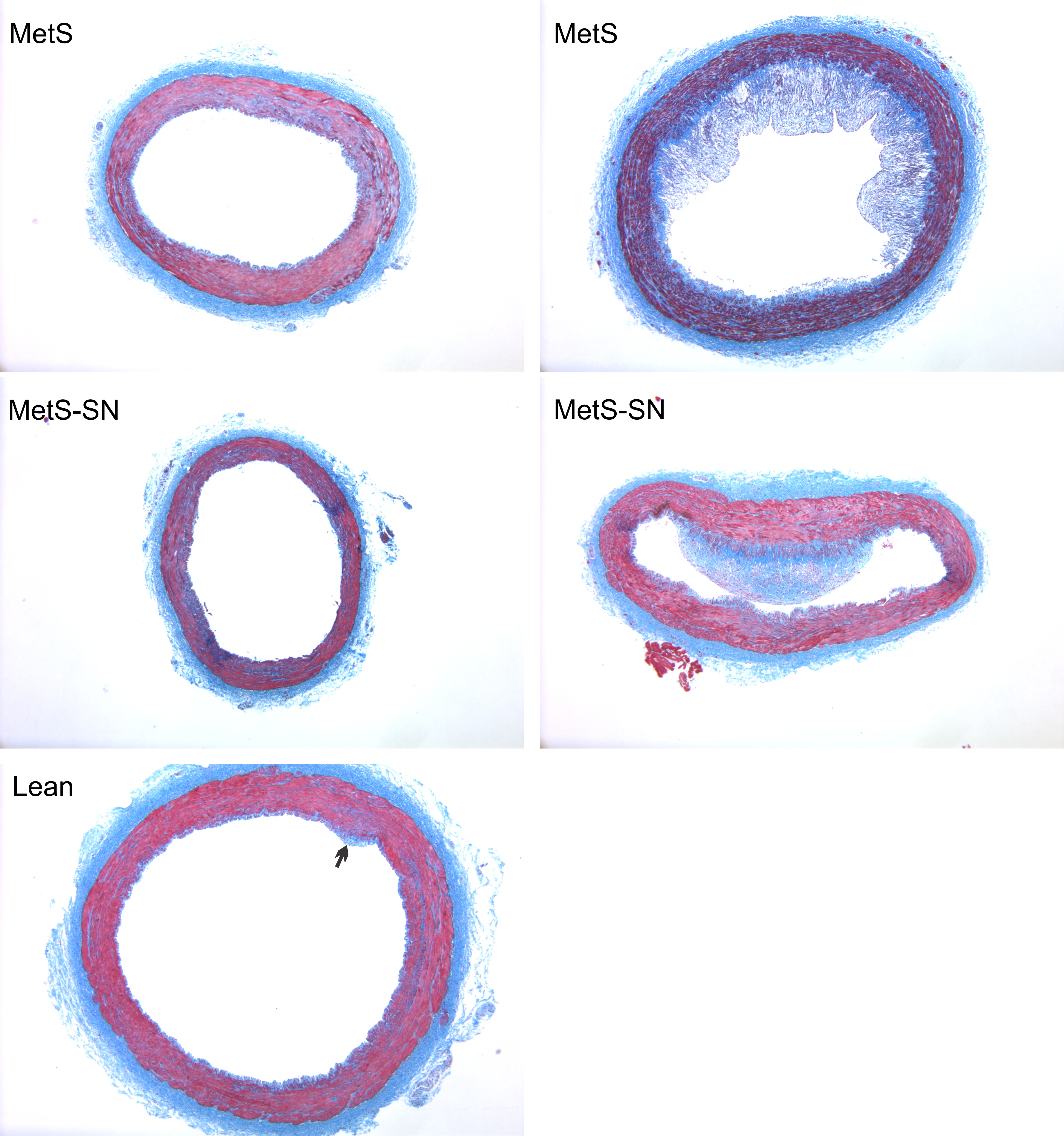

Supplement: Supplementary file 1 — Suppl. Figure 1 Images of the coronary artery sections shown in Fig. 1c-g, but taken with a 4-x objective. The black arrow indicate the small atheroma in a coronary artery ring from a Lean pig (TIFF 10645 kb) [file 395_2017_643_MOESM1_ESM.tif]

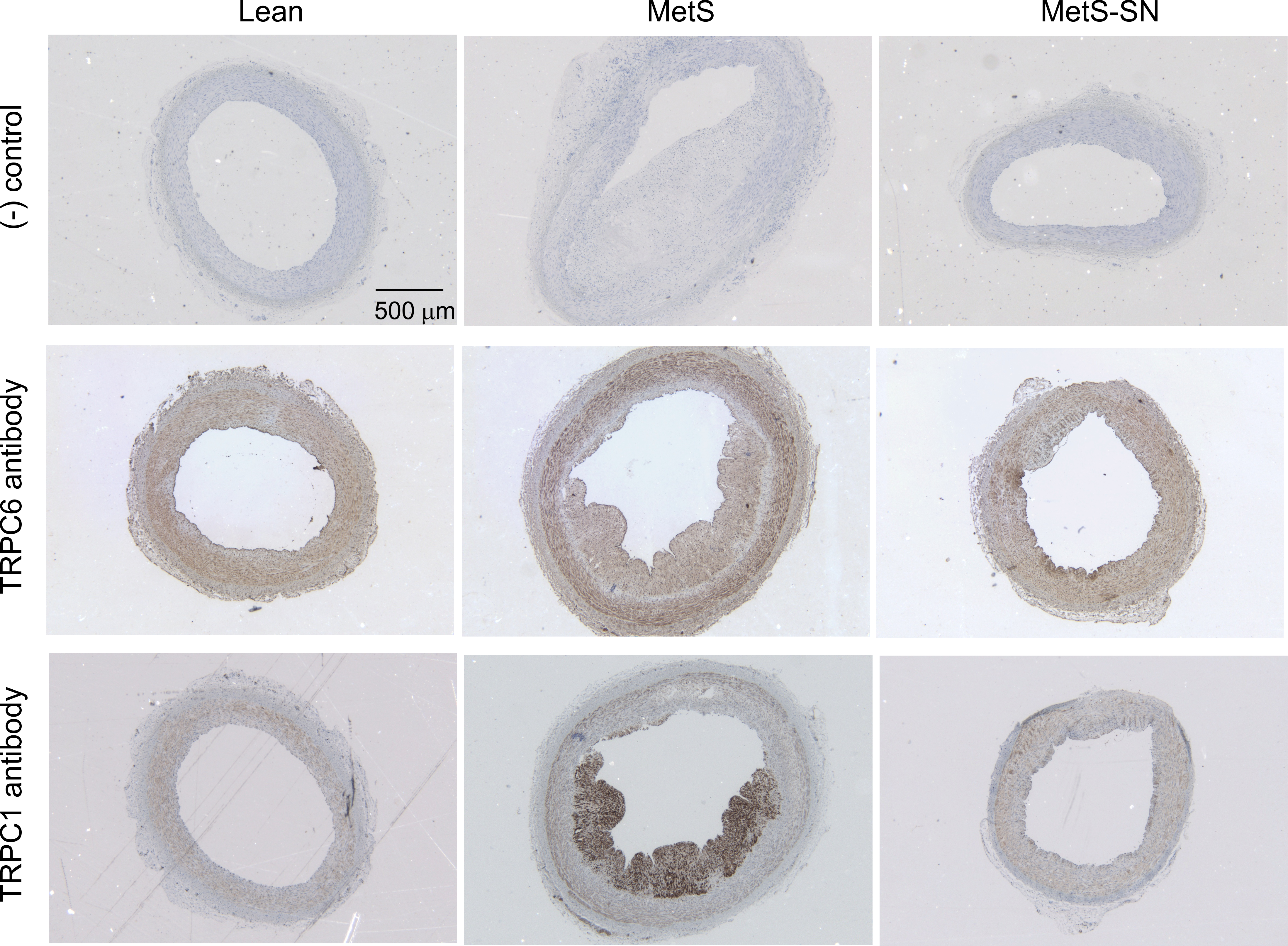

Supplement: Supplementary file 2 — Suppl. Figure 2 Images of the coronary artery sections shown in Fig. 4a, but taken with 4-x objective. The MetS-TRPC1 antibody image is identical to the image “MetS Pig 2-TRPC1 antibody” in Fig. 9 (TIFF 17152 kb) [file 395_2017_643_MOESM2_ESM.tif]

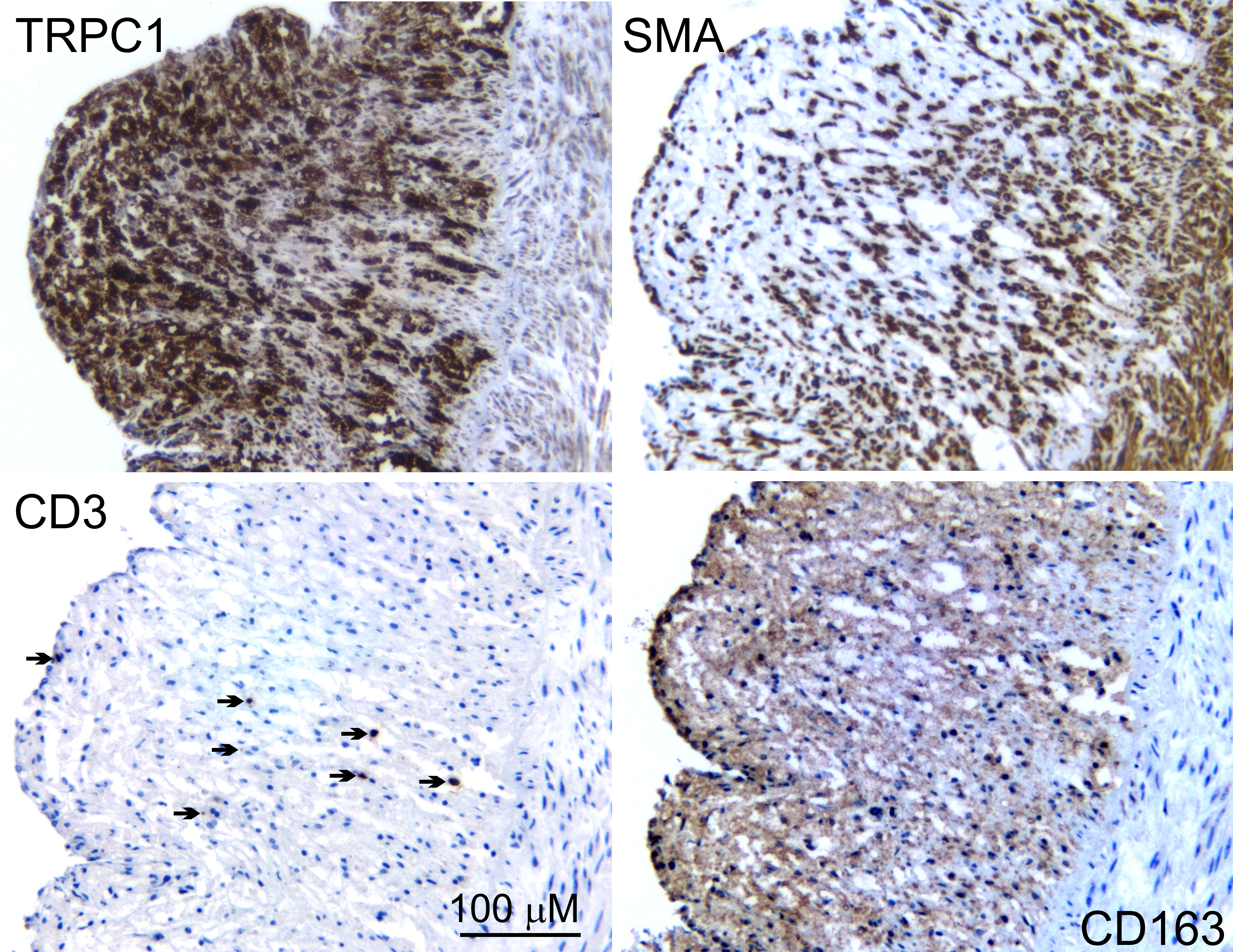

Supplement: Supplementary file 3 — Suppl. Figure 3 Adjacent coronary artery sections from the same MetS pig stained with the TRPC1, α-SMA, CD3, or CD163 antibodies (TIFF 30617 kb) [file 395_2017_643_MOESM3_ESM.tif]

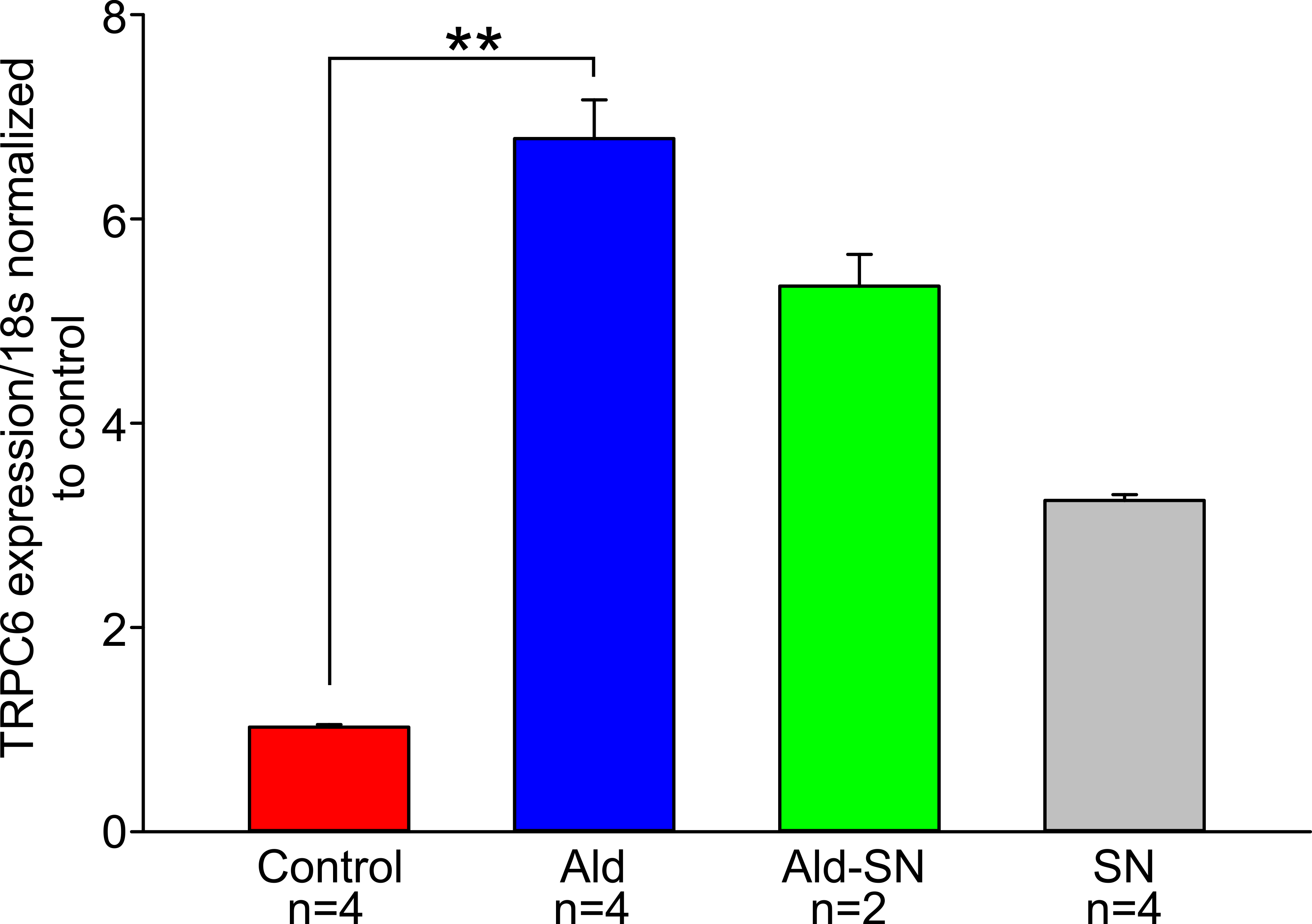

Supplement: Supplementary file 4 — Suppl. Figure 4 Quantitative RT-PCR analysis of TRPC6 expression in organ-cultured Lean pig coronary artery rings. The rings were cultured for 36 h in the presence of either the vehicle, aldosterone (100 nM), aldosterone (100 nM) plus spironolactone (1 μM), or spironolactone (1 μM). The TRPC6 expression was normalized to the 18S rRNA expression level and then to the vehicle control. P = 0.007. The Kruskal–Wallis One-Way Analysis of Variance on Ranks test followed by the post hoc all pairwise multiple comparison Dunn’s test (TIFF 620 kb) [file 395_2017_643_MOESM4_ESM.tif]

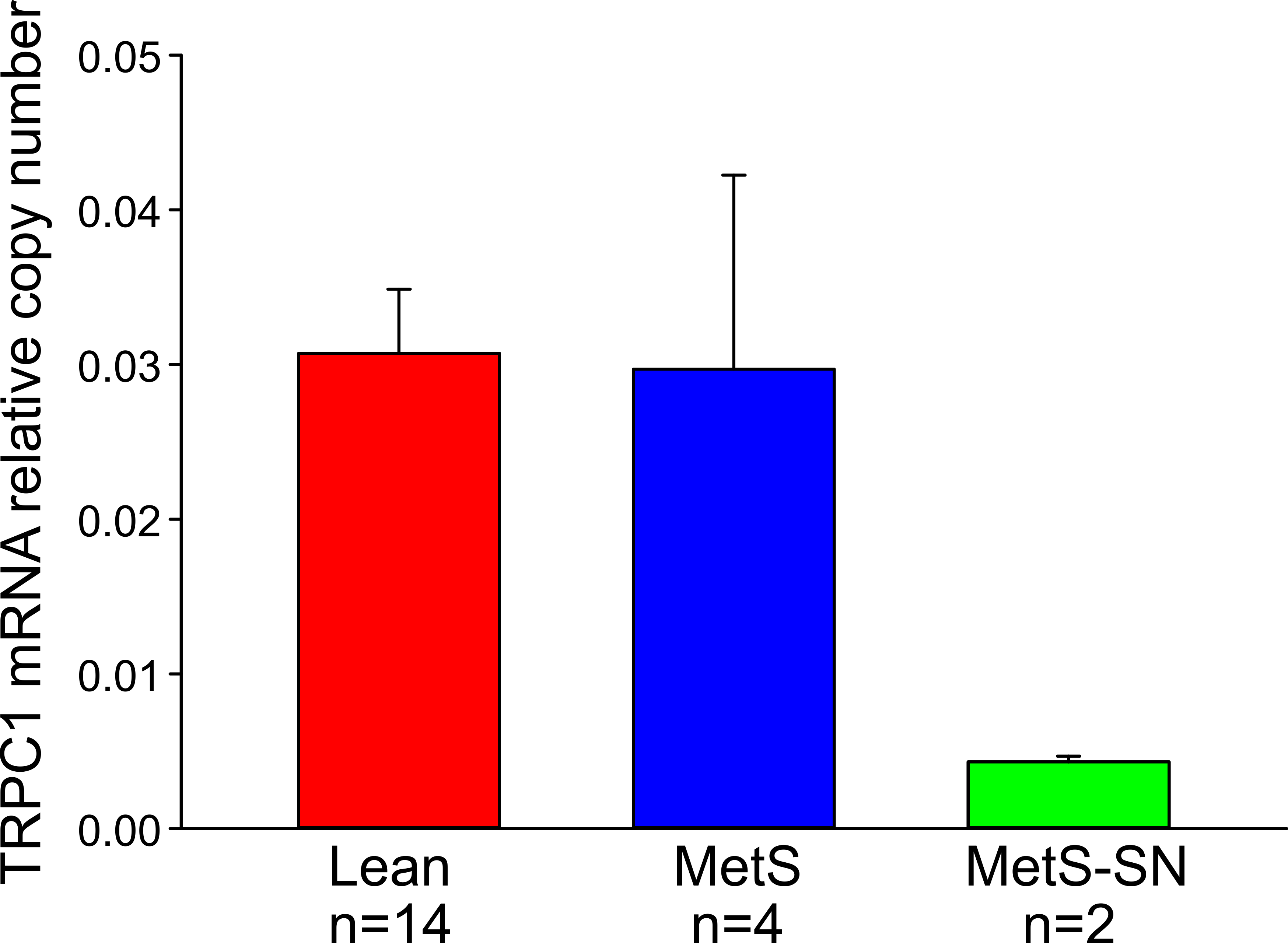

Supplement: Supplementary file 5 — Suppl. Figure 5 Quantitative RT-PCR analysis of TRPC1 expression in the cultured macrophages differentiated from peripheral blood mononuclear cells. Total mRNA was isolated from the cultured macrophage and then reverse-transcribed into cDNA. The primer pairs were purchased from Qiagen. The TRPC1 expression was normalized to the β2 microglobulin expression level. No significant difference was observed among the treatment groups (P = 0.073, the Kruskal–Wallis One-Way Analysis of Variance on Ranks test) (TIFF 641 kb) [file 395_2017_643_MOESM5_ESM.tif]
